# Supplementary material for: Phosphorylation of TFCP2L1 by CDK1 is required for stem cell pluripotency and bladder carcinogenesis
Source: EMBO Mol Med. 2019 Nov 11;12(1):e10880. doi: 10.15252/emmm.201910880 (PMC6949511; doi:10.15252/emmm.201910880)
Supplement: Supplementary file 1 — Appendix [file EMMM-12-e10880-s001.pdf]

# Phosphorylation of TFCP2L1 by CDK1 is required for stem cell pluripotency and bladder carcinogenesis

## Appendix Materials

Heo et al.

**This PDF file includes:**

Appendix Figures S1 to 9

Appendix Tables S1 to 9

# Appendix Figures

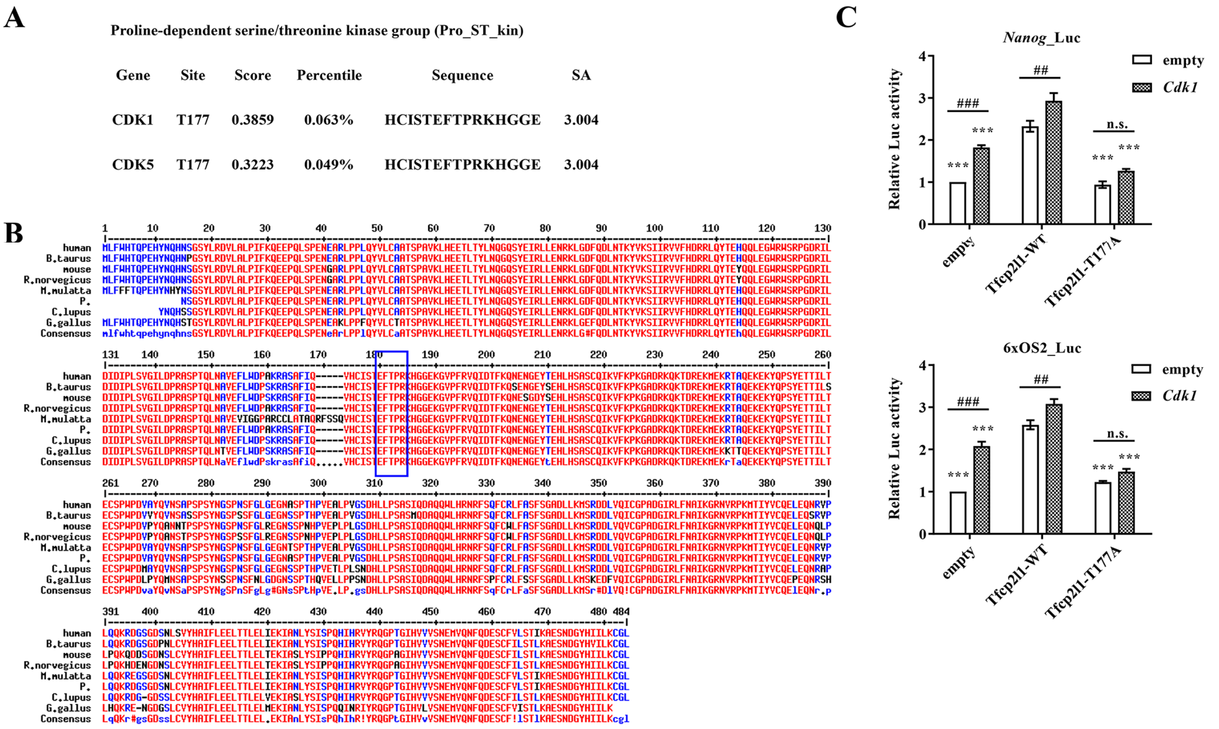


**Appendix Figure S1. Phosphorylation of Tfcp2l1 at Thr177 by CDK1**.

**(A)** *In silico* analysis of phosphorylation of murine Tfcp2l1 protein. **(B)** Sequence alignment of TFCP2L1 sequences from different species. Phosphorylated Thr177 (T177) residue is boxed. **(C)** Promoter activity of murine *Nanog* promoter (upper panel) or 6×OS2 (bottom panel) luciferase (Luc) reporter constructs in mESCs after transient transfection of *Tfcp2l1*-WT (wild type) or *Tfcp2l1*-T177A with (filled box) or without (open box) *Cdk1* ORF ($n = 4$ biological replicates). Data are means ± SEM, ****p* <0.001 compared with Tfcp2l1-WT; ##*p* <0.01, ###*p* <0.001, n.s. = non-significant; one-way or two-way ANOVA with Bonferroni post-hoc tests.

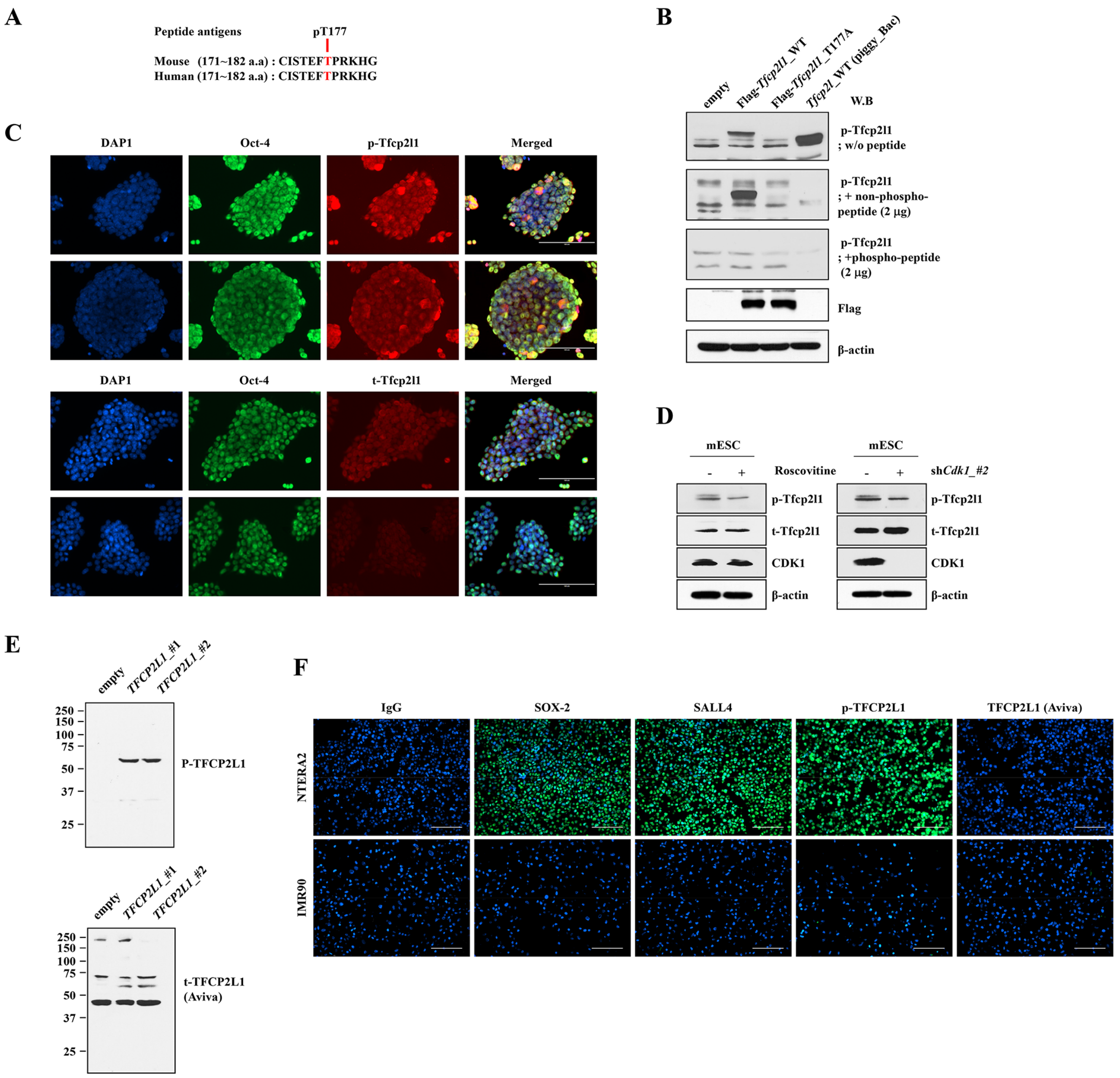


**Appendix Figure S2. Characterization of an In-house Polyclonal Antibody Specific to p-TFCP2L1(T177).**

**(A)** Peptide antigens representing murine and human TFCP2L1 amino acid sequences are shown with T177 modified by phosphorylation shown in red. **(B)** Western blot analysis of mESCs transfected with the indicated Flag-tagged or non-tagged Tfcp2l1-expressing plasmids with in-house polyclonal antibody specific to TFCP2L1 phosphorylated at Thr177 [p-TFCP2L1(T177)]. Specificity of p-TFCP2L1(T177) was validated by competition assay using

2 μg phospho-peptide antigen or non-phospho-peptide antigen. Expression of the indicated Tfcp2l1 proteins was validated using Flag-specific antibody. β-actin was used as an internal control. W.B., Western blot. **(C)** Representative images of immunofluorescence staining for Oct-4 (green) and total (t-Tfcp2l1; antibody purchased from Aviva Systems Biology; OAAB09732; red) or phosphorylated Tfcp2l1 (p-Tfcp2l1; in-house antibody; red) proteins in mESCs (upper panel; ×400 magnification, scale bar = 100 μm, lower panel; ×400 magnification, scale bar = 100 μm). Nuclei were counterstained with DAPI (blue). **(D)** Western blot analysis of mESCs in the treatment of 25 μM roscovitine for 5 h (to inhibit CDK1) or transient expression of shRNA for *Cdk1* (sh*Cdk1*). Note that the inteference of the activity or expression of CDK1 reduced the level of p-Tfcp2l1. **(E)** Western blot analysis of 293 FT cells transfected with human *TFCP2L1*-expressing plasmids (two independent clones; C1 and C2) with in-house p-Tfcp2l1 antibody or commercially available antibody (OAAB09732). **(F)** Immunohistochemistry staining (×200 magnification. scale bars = 100 μm) of p-TFCP2L1, SOX-2, SALL4 stem cell marker proteins in cell-pellet blocks prepared from pluripotent human teratocarcinoma cells (NTERA2) and differentiated primary fibroblasts (IMR90). All antibodies used in tissue microarray (TMA) IHC staining were specific to antigens from NTERA2, and a commercially available TFCP2L1 antibody (Aviva Systems Biology; OAAB09732) was excluded because of low signal in NTERA2 staining.

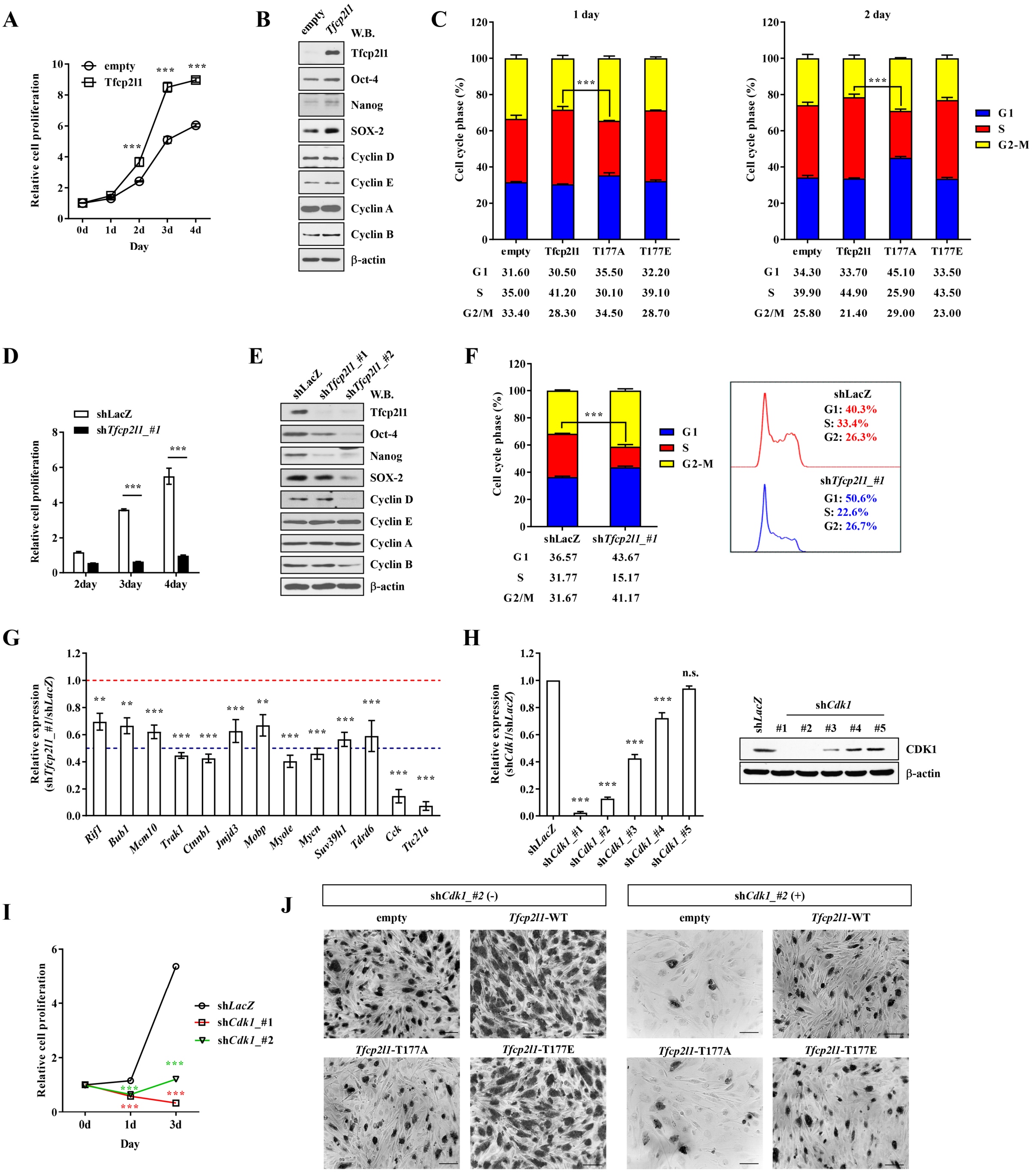


**Appendix Figure S3. Role of Tfcp2l1 in Pluripotency, Proliferation, and Cell Cycle Progression of mESCs**.

**(A, B)** Comparisons of mESCs with and without ectopic expression of *Tfcp2l1*. **(A)** Cell proliferation ($n$ = 8 biological replicates). **(B)** Western blots of proteins related to pluripotency

and the cell cycle. β-actin was used as the loading control. **(C)** Proportions of mESCs in different cell cycle phases at 1 (left panel) and 2 (right panel) days in mESCs infected with *TFCP2L1*-WT, T177A, T177E, or empty control constructs ($n = 3$ biological replicates). The proportions of cells in each cell cycle phase 3 days after infection are reported in the main text as **Fig 1K**. **(D, E, F, G)** Comparisons of mESCs with (sh*Tfcp2l1*) and without (shLacZ) shRNA silencing of *Tfcp2l1* expression **(D)** Cell proliferation ($n = 5$ biological replicates). **(E)** Western blots (two independent shRNAs; #1 and #2). **(F)** FACS analysis of DNA content to determine the cell cycle phase of the mESCs ($n = 3$ biological replicates). **(G)** Real-time qPCR analysis ($n = 4$ biological replicates) of Tfcp2l1 transcription targets related to the cell cycle in mESCs infected with sh*Tfcp2l1*expressing lentiviruses. Lentivirus containing shLacZ or no inserted coding sequence (empty) was used as a control. **(H)** Western blot (upper panel) and real-time qPCR (lower panel) analysis of *Cdk1*-silenced (sh*Cdk1*) mESCs. Five independent sh*Cdk1* constructs were used ($n = 4$ biological replicates). **(I)** Cell viability of mESCs carrying the indicated sh*Cdk1* constructs was determined by the MTT assay on the indicated days ($n = 4$ biological replicates). **(J)** Representative images of AP staining (×200 magnification. scale bars = 200 μm) of mESC colonies infected with the empty control (left panel) or sh*Cdk1* (right panel) construct, which were rescued by overexpression of *Tfcp2l1*-WT, *Tfcp2l1*-T177A, or *Tfcp2l1*-T177E. All quantitative data are mean ± SEM. $**p<0.01$, $***p<0.001$, one-way or two-way ANOVA with the Bonferroni post-test.

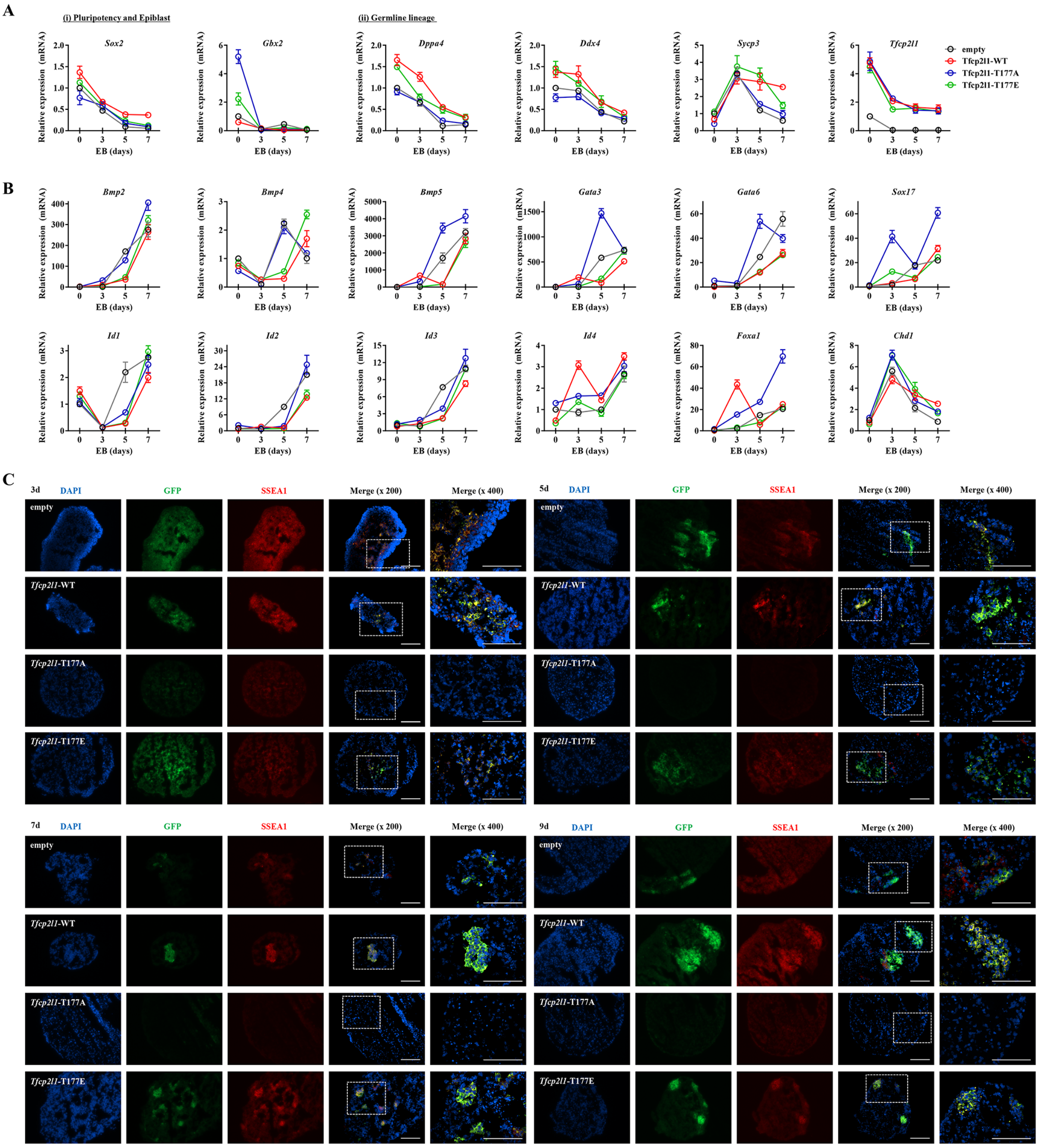


**Appendix Figure S4. Role of Tfcp2l1 Thr177 Phosphorylation on mESC Differentiation.**

**(A, B)** Real-time qPCR analysis of pluripotency- and germline-specific genes **(A)** as well as Tfcp2l1 transcription targets related to differentiation **(B)** in embryoid bodies (EBs). mESCs infected with lentiviruses containing Tfcp2l1-WT (wild-type), T177A, or T177E constructs were used for EB formation. Expression levels are represented as the ratio to 0-day-old EBs

of the empty-control group (means ± SEM, *n* = 4 biological replicates). **(C)** Immunostaining of SSEA-1 protein (red) in Oct4+ (green) germline cells in the 3 day (3d), 5 day (5d), 7 day (7d), and 9 day (9d)-old embryoid bodies (EBs) obtained from gcOct4-GFP ESCs infected with the Tfcp2l1-WT, T177A, and T177E lentivirus (×200 magnifications, scale bar = 100 μm). A merged image with higher magnification (×400 magnifications, scale bar = 100 μm) is shown in the right panel. Nuclei were counterstained with DAPI (blue).

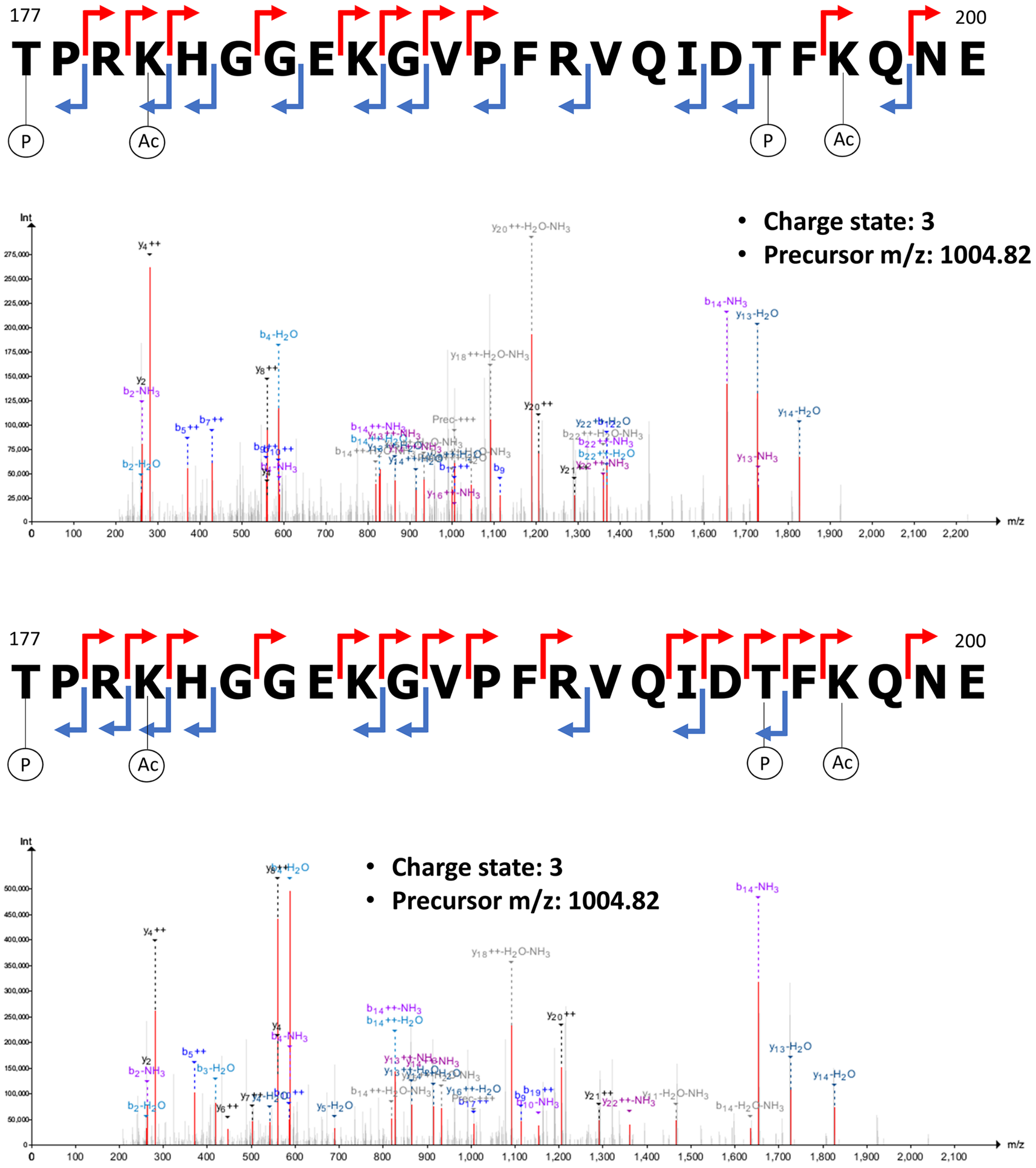


**Appendix Figure S5. Thr177 Phosphorylation of TFCP2L1 in Human Bladder Cancer Cells.**

FLAG-tagged TFCP2L1 phosphorylated residues in chymotrypsin digests of FLAG-immunoprecipitates (IP) of a T24 bladder cancer cell line expressing FLAG-tagged TFCP2L1

were identified by mass spectrometry analysis. Two representative MS/MS spectra of the Thr177 phosphorylated peptide containing other post-translational modifications are depicted. Red and blue lines in the peptide fragmentation map indicate y ions and b ions, respectively. Red lines on MS/MS spectrum indicating matched fragment ions and adduct ions such as ammonium and water are shown in purple and sky-blue. Charge states and precursor m/z are shown in the text box. Numbers on peptides show amino acid residue numbers and letters “P” and “Ac” in a circle indicate phosphorylation and acetylation, respectively.

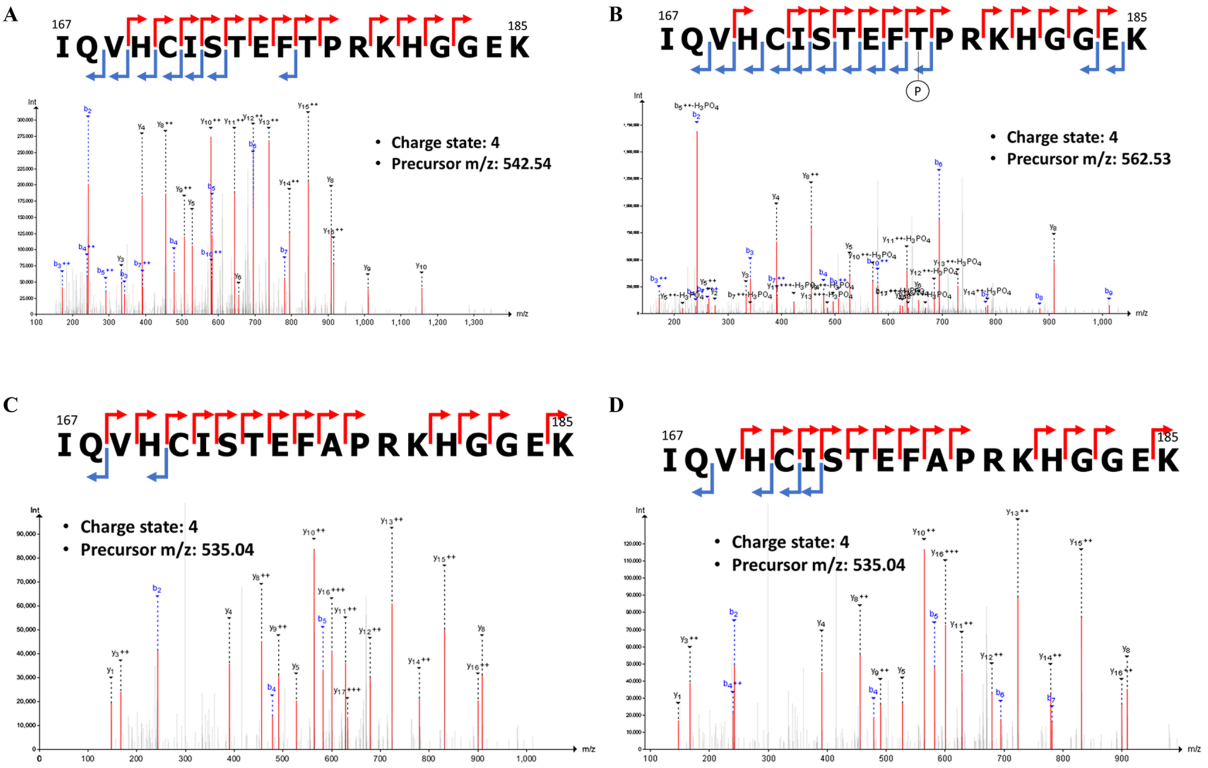


**Appendix Figure S6. *In vitro* Kinase Assay for Thr177 Phosphorylation of TFCP2L1 by CDK1.**

**(A – D)** Human CDK1/Cyclin B recombinant proteins were used for *in vitro* kinase reactions containing the TFCP2L1 Thr177 peptide **(A and B**) and Thr177A mutant peptide **(C and D)**. Thr177 phosphorylation of the indicated peptides were identified by mass spectrometry analysis. **(A and C)** Reaction products in the absence of the human CDK1/Cyclin B complex were used as a control. Representative MS/MS spectra of the Thr177 phosphorylated peptide and corresponding peptide sequence are depicted. Red and blue lines in the peptide fragment map indicate y ions and b ions, respectively, and red lines in the MS/MS spectrum indicate matched fragment ions from the precursor peptide ion. Numbers on the peptide fragment map are amino acid residue numbers.

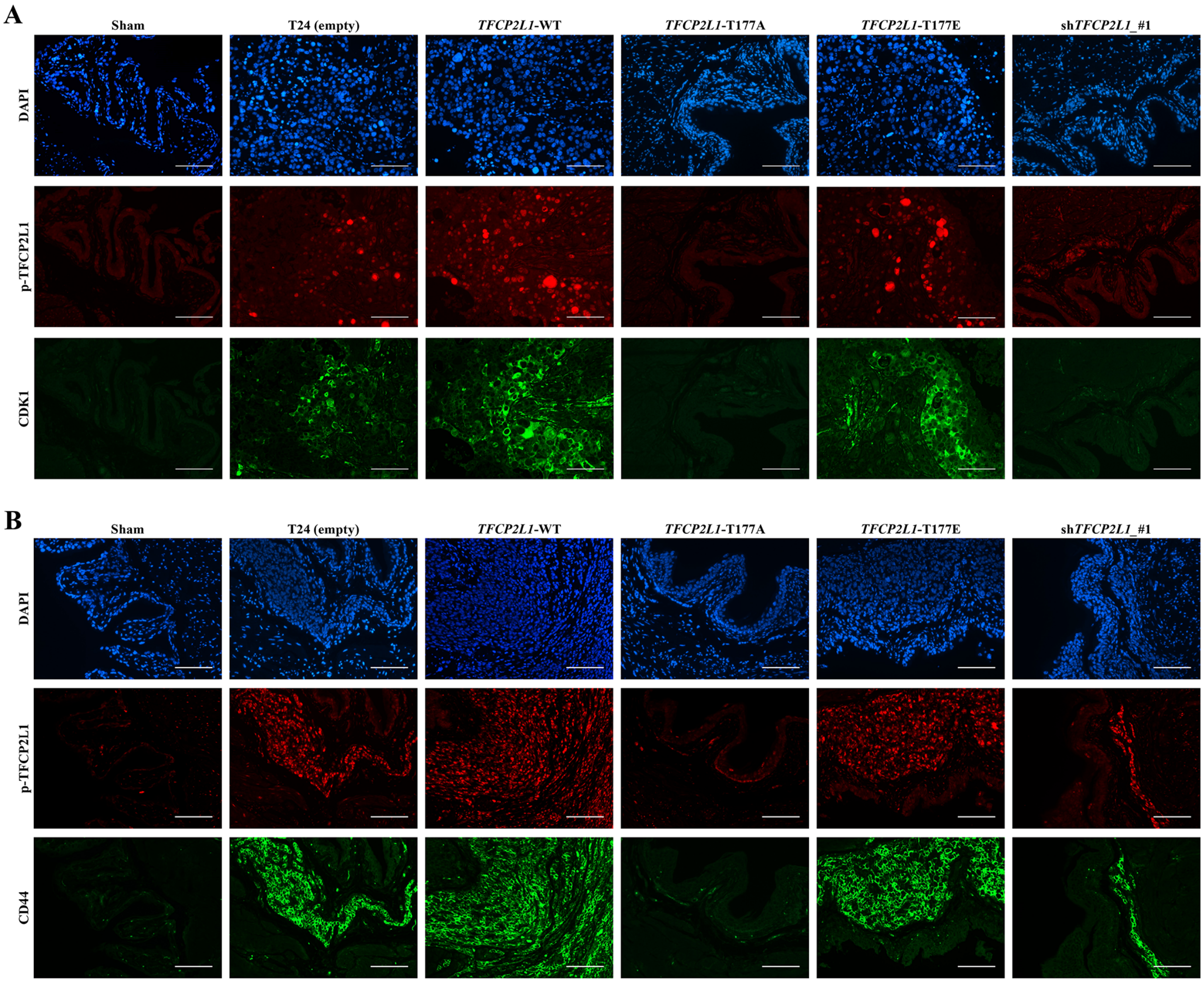


**Appendix Figure S7. Immunostaining of p-TFCP2L1 and CDK1 Proteins in Xenograft Samples.**

**(A, B)** Immunofluorescence assay for detecting p-TFCP2L1 (red) and CDK1 (green; **A**) or p-TFCP2L1 (red) and CD44 (green; **B**) in the tumors from orthotopically injected T24 cells infected with lentiviruses expressing the indicated *TFCP2L1* ORFs, shRNA (sh*TFCP2L1*), or empty control constructs. Representative images are shown at ×200 magnification. Scale bars = 100 μm. Nuclei were stained with DAPI (blue). The merged images are shown in the main text as **Figs 7E and F**.

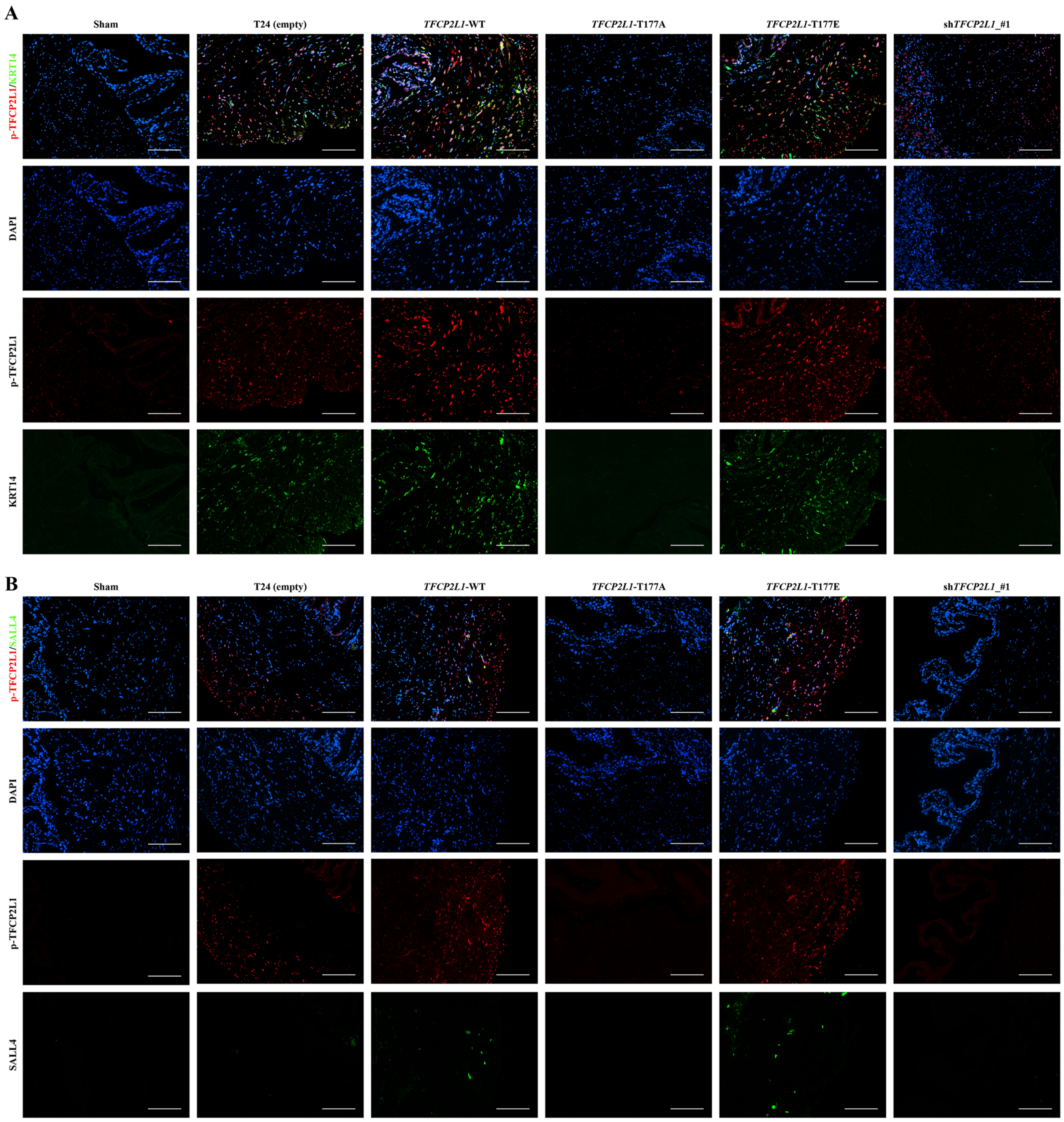


**Appendix Figure S8. Immunostaining of Bladder Cancer Stem Cell Markers and CDK1 Proteins in Xenograft Samples.**

**(A, B)** Immunofluorescence of p-TFCP2L1 (red) and bladder cancer stem cell markers KRT14 (green; **A**) and SALL4 (green; **B**) in tumors established by orthotopically injecting T24 cells infected with lentiviruses expressing the indicated *TFCP2L1* ORFs, shRNA (sh*TFCP2L1*), or infected with the empty control construct. Representative images are shown at ×200 magnification. Scale bars = 100 μm. Nuclei were stained with DAPI (blue).

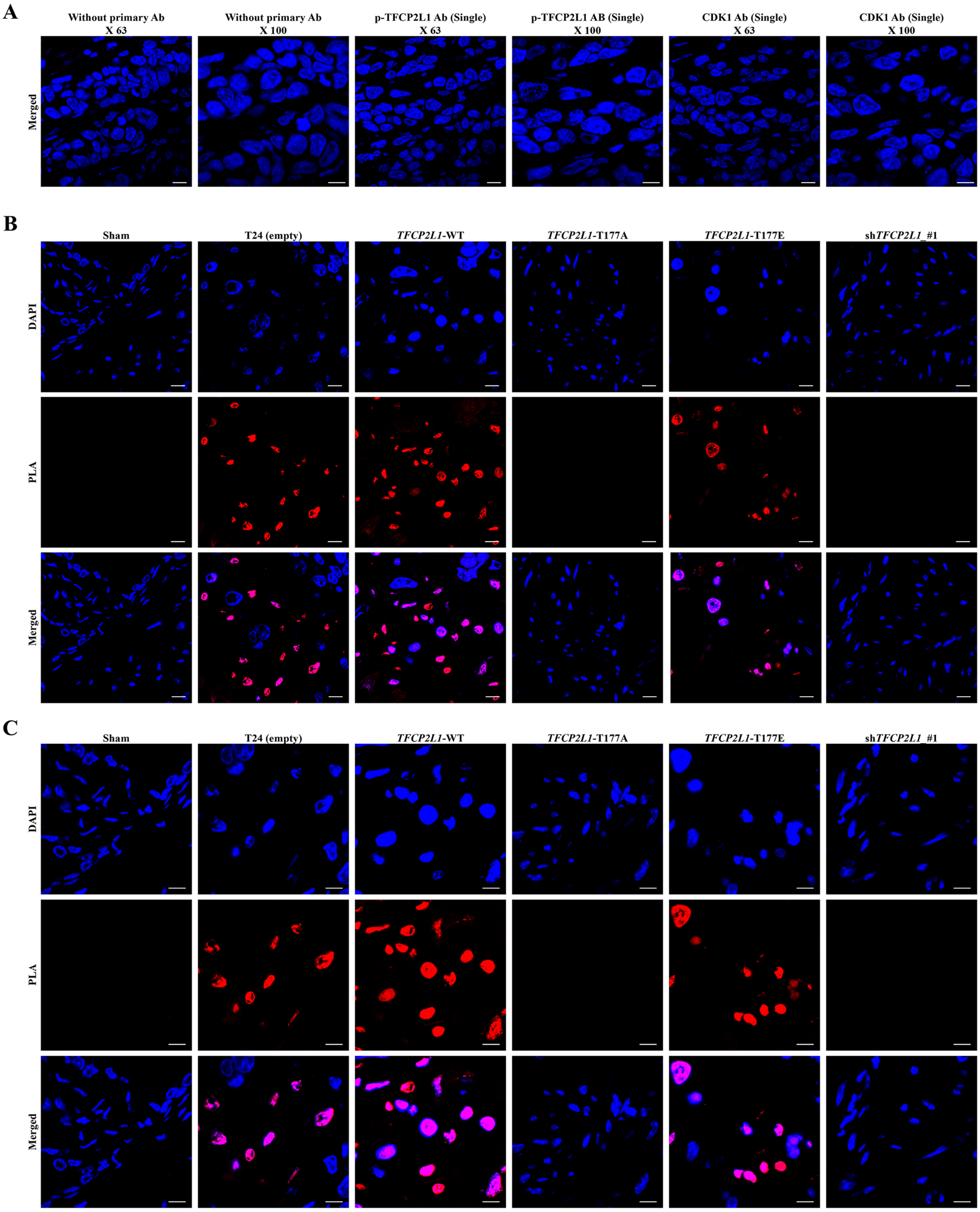


**Appendix Figure S9. Proximity Ligation Assay (PLA) of Xenograft Samples.**

**(A)** To rule out the possibility of non-specific staining in proximity ligation assay (PLA) assay, bladder tissues of mice transplanted with T24 cells harboring the empty control

construct were stained with single anti-p-TFCP2L1 or CDK1 antibody or without primary antibodies. Representative images are shown at ×630 (left panel) or ×1,000 (right panel) magnification. Scale bars = 20 μm. **(B, C)** PLA staining for detecting colocalization of p-TFCP2L1 and CDK1 proteins in xenograft tumors derived from T24 cells harboring *TFCP2L1*-WT (wild-type), T177A, or T177E constructs, shRNA (sh*TFCP2L1*), or empty control constructs. Representative confocal microscopic images are shown at ×630 **(B)** or ×1,000 **(C)** magnification. Scale bars = 20 μm.

## Appendix Tables

**Appendix Table 1. List of Genes and Gene Ontology Terms in which Tfcp2l1 DNA Binding are Affected by Thr177 Phosphorylation.**

| | Primer location | T177A | G2/M | Gene | Gene Ontology |
|---|---|---|---|---|---|
| T177A (↓) & G2/M (↑) | 7 | ↓ | ↑ | *Myo1e* | Actin filament polymerization |
| | 16 | ↓ | ↑ | *Xirf1* | Negatvie regulation of cell proliferattion |
| | 22 | ↓ | ↑ | *LF219113* | Polycomb-Associated Non-Coding RNAs |
| | 29 | ↓ | ↑ | *Fndc5* | positive regulation of brown fat cell differentiation |
| | 36 | ↓ | ↑ | *Porcn* | Wnt signaling pathway |
| | 45 | ↓ | ↑ | *Map6* | microtubule cytoskeleton organization |
| T177A (↓) & G2/M (not affected) | 1 | ↓ | n.a. | *Dusp13* | Meiotic cell cycle |
| | 1 | ↓ | n.a. | *Samd8* | lipid metabolic process |
| | 21 | ↓ | n.a. | *Idua* | Carhohydrate metabolic process |
| | 21 | ↓ | n.a. | *Slc26a1* | Biocarbonate transport |
| | 21 | ↓ | n.a. | *Dgkq* | cAMP-mediated signaling |
| | 23 | ↓ | n.a. | *Lnpep* | proteolysis |
| | 27 | ↓ | n.a. | *C2cd4a* | calcium ion-dependent exocytosis |
| | 27 | ↓ | n.a. | *Vps13c* | mitochondrion organization |
| | 28 | ↓ | n.a. | *Wdr13* | negative regulation of type B pancreatic cell proliferation |
| | 28 | ↓ | n.a. | *Rbm3* | positive regulation of translation |
| | 35 | ↓ | n.a. | *D630039A03Rik* | biological_process |
| | 39 | ↓ | n.a. | *Slc25a27* | positive regulation of cell proliferation |
| | 39 | ↓ | n.a. | *Cyp39a1* | cholesterol catabolic process |
| | 40 | ↓ | n.a. | *Foxn3* | Cell cycle |
| | 41 | ↓ | n.a. | *Cyp26a1* | central nervous system development |
| | 44 | ↓ | n.a. | *Fgf1* | cell proliferation |
| | 46 | ↓ | n.a. | *Lrrc61* | Endometrial Neoplasms |
| * | 47 | ↓ | ↓ | *Ctnnb1* | Wnt signaling pathway |
| T177A (not affected) & G2/M (↑) | 5 | n.a. | ↑ | *Mycl* | Cell proliferation |
| | 10 | n.a. | ↑ | *Cpox* | Heme biosynthetic process |
| | 11 | n.a. | ↑ | *Carf* | Cellular response to calcium ion |
| | 38 | n.a. | ↑ | *Hyal1* | positive regulation of G1/S transition of mitotic cell cycle |
| | 38 | n.a. | ↑ | *Hyal2* | hematopoietic progenitor cell differentiation, |
| | 38 | n.a. | ↑ | *Hyal3* | carbohydrate metabolic process |
| | 38 | n.a. | ↑ | *Ifrd2* | biological_process |
| | 38 | n.a. | ↑ | *Nat6* | positive regulation of G1/S transition of mitotic cell cycle |
| T177A (not affected) & G2/M (↓) | 8 | n.a. | ↓ | *Muc5b* | O-glycan processing |
| | 12 | n.a. | ↓ | *Foxj3* | cell differentiation |
| | 12 | n.a. | ↓ | *Guca2a* | positive regulation of guanylate cyclase activity |
| | 17 | n.a. | ↓ | *Syn3* | Neurotransmitter secretion |
| | 20 | n.a. | ↓ | *Fam43a* | n.d. |
| | 20 | n.a. | ↓ | *Lsg1* | nuclear export |
| | 30 | n.a. | ↓ | *Sqle* | cholesterol metabolic process |
| | 37 | n.a. | ↓ | *Slc6a20a* | amino acid transport |

The affected DNA binding of Tfcp2l1 by Thr177 (T177) phosphorylation or G2/M cell cycle is based on qChIP results of the validated Tfcp2l1 targets (primer locations T1–T47; see also **Figs EV2C and D**). Genes located within 10 kb upstream or downstream from the affected Tfcp2l1 target sites were listed and characterized by the Gene Ontology (GO) terms. Increased (↑) and decreased (↓) DNA binding of Tfcp2l1 are indicated. n.a., not affected. GO terms related to cell cycle, proliferation, and differentiation processes were marked in red. *: T177A (↓) & G2/M (↓).

**Appendix Table 2. Demographic Information for the 400 Cases of Bladder Cancer.**

| Parameters | | Numbers | % |
|---|---|---|---|
| Age (years) | < 70 | 186 | 46.5 |
| | ≥ 70 | 214 | 53.5 |
| Sex | Male | 346 | 86.5 |
| | Female | 54 | 13.5 |
| Size | <1 cm | 49 | 12.3 |
| | 1 - 2 cm | 150 | 37.5 |
| | > 2 cm | 153 | 38.3 |
| | Not assessable† | 48 | 12.0 |
| Multiplicity | Unifocal | 199 | 49.8 |
| | Multifocal | 201 | 50.3 |
| Grade | PUNLMP | 21 | 5.3 |
| | Low | 116 | 29.0 |
| | High | 263 | 65.8 |
| Lymphovascular invasion | Absent | 328 | 82.0 |
| | Present | 72 | 18.0 |
| Carcinoma in situ | Absent | 276 | 69.0 |
| | Present | 124 | 31.0 |
| pT category | Ta | 109 | 27.3 |
| | T1 | 163 | 40.8 |
| | T2-T4 | 122 | 30.5 |
| | Not assessable* | 6 | 1.5 |
| Muscularis propria invasion | Absent | 272 | 68.0 |
| | Present | 122 | 30.5 |
| | Not assessable* | 6 | 1.5 |
| Lymph node metastasis | Absent | 123 | 30.8 |
| | Present | 52 | 13.0 |
| | Not assessable† | 225 | 56.3 |
| Tumor recurrence | Absent | 260 | 65.0 |
| | Present | 140 | 35.0 |
| Distance metastasis | Absent | 352 | 88.0 |
| | Present | 48 | 12.0 |
| Cancer-specific death | Alive | 248 | 62.0 |
| | Dead | 152 | 38.0 |
| p-TFCP2L1 | Low expression | 221 | 55.3 |
| | High expression | 179 | 44.8 |
| CDK1 | Low expression | 215 | 53.8 |
| | High expression | 185 | 46.3 |
| Co-expression | Low proportion | 193 | 48.3 |
| | High proportion | 207 | 51.7 |

Values are presented as *n* (%). †Not assessable because clinical information was not available. *Not assessable because of cautery artifact, fragmentation, or incorrect orientation of tumor tissues. PUNLMP; papillary urothelial neoplasm with low malignant potential.

**Appendix Table 3. Correlation Between Clinicopathological Factors and Cancer-Specific Survival.**

| Parameter | | Cancer-specific survival | | |
|---|---|---|---|---|
| | | Alive (n=248) | Dead (n=152) | P-Value |
| Age (years) | < 70 | 141 (75.8) | 45 (24.2) | <0.001 |
| | ≥ 70 | 107 (50.0) | 107 (50.0) | |
| Sex | Male | 215 (62.1) | 131 (37.9) | 0.885 |
| | Female | 33 (61.1) | 21 (38.9) | |
| Size | <1 cm | 39 (79.6) | 10 (20.4) | <0.001 |
| | 1 - 2 cm | 105 (70.0) | 45 (30.0) | |
| | > 2 cm | 82 (53.6) | 71 (46.4) | |
| | Not assessable† | 22 (45.8) | 26 (54.2) | |
| Multiplicity | Unifocal | 135 (67.8) | 64 (32.2) | 0.017 |
| | Multifocal | 113 (56.2) | 88 (43.8) | |
| Grade | PUNLMP | 19 (90.5) | 2 (9.5) | <0.001 |
| | Low | 94 (81.0) | 22 (19.0) | |
| | High | 135 (51.3) | 128 (48.7) | |
| Lymphovascular invasion | Absent | 227 (69.2) | 101 (30.8) | <0.001 |
| | Present | 21 (29.2) | 51 (70.8) | |
| Carcinoma in situ | Absent | 179 (64.9) | 97 (35.1) | 0.079 |
| | Present | 69 (55.6) | 55 (44.4) | |
| pT category | Ta | 94 (86.2) | 15 (13.8) | <0.001 |
| | T1 | 109 (66.9) | 54 (33.1) | |
| | T2-T4 | 40 (32.8) | 82 (67.2) | |
| | Not assessable* | 5 (83.3) | 1 (16.7) | |
| Muscularis propria invasion | Absent | 203 (74.6) | 69 (25.4) | <0.001 |
| | Present | 40 (32.8) | 82 (67.2) | |
| | Not assessable* | 5 (83.3) | 1 (16.7) | |
| Lymph node metastasis | Absent | 76 (61.8) | 47 (38.2) | <0.001 |
| | Present | 12 (23.1) | 40 (76.9) | |
| | Not assessable† | 160 (71.1) | 65 (28.9) | |
| Tumor recurrence | Absent | 162 (62.3) | 98 (37.7) | 0.863 |
| | Present | 86 (61.4) | 54 (38.6) | |
| Distance metastasis | Absent | 242 (68.8) | 110 (31.3) | <0.001 |
| | Present | 6 (12.5) | 42 (87.5) | |
| p-TFCP2L1 | Low expression | 153 (69.2) | 68 (30.8) | 0.001 |
| | High expression | 95 (53.1) | 84 (46.9) | |
| CDK1 | Low expression | 149 (69.3) | 66 (30.7) | 0.001 |
| | High expression | 99 (53.5) | 86 (46.5) | |
| Co-expression | Low proportion | 137 (71.0) | 56 (29.0) | <0.001 |
| | High proportion | 111 (53.6) | 96 (46.4) | |

Values are shown as *n* (%). †Not assessable because clinical information not available. *Not assessable because of cautery artifact, fragmentation, or incorrect orientation of tumor tissues. PUNLMP; papillary urothelial neoplasm with low malignant potential.

**Appendix Table 4. Correlation Between CDK1-TFCP2L1 Expression and Clinicopathological Factors in Cases of Bladder Cancer with Carcinoma in-situ (CIS).**

| Parameter | | p-TFCP2L1 expression | | | CDK1 expression | | | Co-expression proportion | | |
|---|---|---|---|---|---|---|---|---|---|---|
| | | Low | High | P-value | Low | High | P-value | Low | High | P-value |
| Age (years) | < 70 | 32 | 28 | 0.472 | 32 | 28 | 0.844 | 22 | 38 | 0.328 |
| | % | 53.3% | 46.7% | | 53.3% | 46.7% | | 36.7% | 63.3% | |
| | ≥ 70 | 30 | 34 | | 33 | 31 | | 29 | 35 | |
| | % | 46.9% | 53.1% | | 51.6% | 48.4% | | 45.3% | 54.7% | |
| Sex | Male | 49 | 56 | 0.081 | 56 | 49 | 0.632 | 42 | 63 | 0.548 |
| | % | 46.7% | 53.3% | | 53.3% | 46.7% | | 40.0% | 60.0% | |
| | Fenale | 13 | 6 | | 9 | 10 | | 9 | 10 | |
| | % | 68.4% | 31.6% | | 47.4% | 52.6% | | 47.4% | 52.6% | |
| Size | <1 cm | 7 | 13 | 0.592 | 13 | 7 | 0.231 | 10 | 10 | 0.225 |
| | % | 35.0% | 65.0% | | 65.0% | 35.0% | | 50.0% | 50.0% | |
| | 1 - 2 cm | 20 | 18 | | 24 | 14 | | 15 | 23 | |
| | % | 52.6% | 47.4% | | 63.2% | 36.8% | | 39.5% | 60.5% | |
| | > 2 cm | 23 | 27 | | 29 | 21 | | 17 | 33 | |
| | % | 46.0% | 54.0% | | 58.0% | 42.0% | | 34.0% | 66.0% | |
| | Not assessable† | 12 | 4 | | 12 | 4 | | 9 | 7 | |
| | % | 75.0% | 25.0% | | 75.0% | 25.0% | | 56.3% | 43.8% | |
| Multiplicity | Unufocal | 27 | 21 | 0.269 | 27 | 21 | 0.497 | 23 | 25 | 0.222 |
| | % | 56.3% | 43.8% | | 56.3% | 43.8% | | 47.9% | 52.1% | |
| | Multifocal | 35 | 41 | | 38 | 38 | | 28 | 48 | |
| | % | 46.1% | 53.9% | | 50.0% | 50.0% | | 36.8% | 63.2% | |
| Grade | Punlmp | 0 | 0 | 0.649 | 0 | 0 | 0.209 | 0 | 0 | 0.329 |
| | % | 0.0% | 0.0% | | 0.0% | 0.0% | | 0.0% | 0.0% | |
| | Low | 3 | 2 | | 4 | 1 | | 1 | 4 | |
| | % | 60.0% | 40.0% | | 80.0% | 20.0% | | 20.0% | 80.0% | |
| | High | 59 | 60 | | 61 | 58 | | 50 | 69 | |
| | % | 49.6% | 50.4% | | 51.3% | 48.7% | | 42.0% | 58.0% | |
| Lymphovasular invasion | Absence | 49 | 40 | 0.073 | 49 | 40 | 0.348 | 38 | 51 | 0.572 |
| | % | 55.1% | 44.9% | | 55.1% | 44.9% | | 42.7% | 57.3% | |
| | Presence | 13 | 22 | | 16 | 19 | | 13 | 22 | |
| | % | 37.1% | 62.9% | | 45.7% | 54.3% | | 37.1% | 62.9% | |
| pT category | Ta | 6 | 6 | 0.528 | 5 | 7 | 0.901 | 3 | 9 | 0.486 |
| | % | 50.0% | 50.0% | | 41.7% | 58.3% | | 25.0% | 75.0% | |
| | T1 | 28 | 24 | | 30 | 22 | | 23 | 29 | |
| | % | 53.8% | 46.2% | | 57.7% | 42.3% | | 44.2% | 55.8% | |
| | T2-4 | 27 | 32 | | 29 | 30 | | 25 | 34 | |
| | % | 45.8% | 54.2% | | 49.2% | 50.8% | | 42.4% | 57.6% | |
| | Not assessable† | 0 | 0 | | 0 | 0 | | 0 | 0 | |
| | % | 0.0% | 0.0% | | 0.0% | 0.0% | | 0.0% | 0.0% | |
| Muscularis propria invasion | Absence | 34 | 30 | 0.415 | 35 | 29 | 0.539 | 26 | 38 | 0.844 |
| | % | 53.1% | 46.9% | | 54.7% | 45.3% | | 40.6% | 59.4% | |
| | Presence | 27 | 32 | | 29 | 30 | | 25 | 34 | |
| | % | 45.8% | 54.2% | | 49.2% | 50.8% | | 42.4% | 57.6% | |
| | Not assessable† | 0 | 0 | | 0 | 0 | | 0 | 0 | |
| | % | 0.0% | 0.0% | | 0.0% | 0.0% | | 0.0% | 0.0% | |
| Lymph node metastasis | Absence | 20 | 29 | 0.843 | 21 | 28 | 0.713 | 15 | 34 | 0.493 |
| | % | 40.8% | 59.2% | | 42.9% | 57.1% | | 30.6% | 69.4% | |
| | Presence | 10 | 16 | | 10 | 16 | | 10 | 16 | |
| | % | 38.5% | 61.5% | | 38.5% | 61.5% | | 38.5% | 61.5% | |
| | Not assessable† | 32 | 17 | | 34 | 15 | | 26 | 23 | |
| | % | 65.3% | 34.7% | | 69.4% | 30.6% | | 53.1% | 46.9% | |
| Tumor recurrence | Absence | 42 | 46 | 0.429 | 45 | 43 | 0.655 | 38 | 50 | 0.468 |
| | % | 47.7% | 52.3% | | 51.1% | 48.9% | | 43.2% | 56.8% | |
| | Presence | 20 | 16 | | 20 | 16 | | 13 | 23 | |
| | % | 55.6% | 44.4% | | 55.6% | 44.4% | | 36.1% | 63.9% | |
| Distance metastasis | Absence | 54 | 50 | 0.329 | 56 | 48 | 0.468 | 46 | 58 | 0.109 |
| | % | 51.9% | 48.1% | | 53.8% | 46.2% | | 44.2% | 55.8% | |
| | Presence | 8 | 12 | | 9 | 11 | | 5 | 15 | |
| | % | 40.0% | 60.0% | | 45.0% | 55.0% | | 25.0% | 75.0% | |
| Cancer-specific death | Alive | 39 | 30 | 0.104 | 41 | 28 | 0.08 | 35 | 34 | 0.015 |
| | % | 56.5% | 43.5% | | 59.4% | 40.6% | | 50.7% | 49.3% | |
| | Dead | 23 | 32 | | 24 | 31 | | 16 | 39 | |
| | % | 41.8% | 58.2% | | 43.6% | 56.4% | | 29.1% | 70.9% | |

Values are shown as *n* and %. †Not assessable because clinical information not available. *Not assessable because of cautery artifact, fragmentation, or incorrect orientation of tumor tissues. PUNLMP; papillary urothelial neoplasm with low malignant potential.

**Appendix Table 5. Information on Primary Antibodies Used in this Study on the Normal Urinary Bladder and Bladder-Cancer Tissues.**

| Antibody | Dilution | Company | Subcellular location |
|---|---|---|---|
| Transcription factor CP2-like protein 1 (TFCP2L1) | 1:5,000 | Home-made (AbFrontier) | Nucleus |
| Cyclin-dependent kinase 1 (CDK1) | 1:200 | Sc-54 (Santa Cruz) | Nucleus & Cytoplasm |
| SALL4 | 1:100 | CM 384 A,C (Biocare) | Nucleus |
| CD44 | 1:100 | M 7082 (Dako) | Membrane & Cytoplasm |
| Cytokeratin | 1:700 | AE1/AE3, M3515 (Dako) | Cytoplasm |

The dilutions and the subcellular location of each antigen are summarized.

**Appendix Table 6. Primers used for ChIP analysis**

| **Fig EV2_C-D** | | |
|---|---|---|
| **Name** | **Sequence** | **Species** |
| TF_qChIP_T1_F | GGGCAGGCTGAGTGGAACAGTCCTAAAG | Mouse |
| TF_qChIP_T1_R | TAGCAGAGACACCAGGGAGAGATGTTAGGC | Mouse |
| TF_qChIP_T2_F | AAACTTGATCACATACCTCTGTTTACCCAG | Mouse |
| TF_qChIP_T2_R | CTCTGTCCAGGAACACAAGCTTCTAGCA | Mouse |
| TF_qChIP_T3_F | TGGCTCATGTGTAATTTGGTCTTAGCAG | Mouse |
| TF_qChIP_T3_R | GGCCTCTCTATGTCACTGTAGTGTATCAAG | Mouse |
| TF_qChIP_T4_F | GGTTCACACAGGCGTGGAGCAGGACACTCA | Mouse |
| TF_qChIP_T4_R | AATATCCTCGCCAGTTCCGGAGCAC | Mouse |
| TF_qChIP_T5_F | TGCAAACCTGTCCTTCTCGAGCAACTT | Mouse |
| TF_qChIP_T5_R | TGTTGGCTGAGTAGCGTTCCTAGAGGTACA | Mouse |
| TF_qChIP_T6_F | GCCATTCTTCCCTTTGCTCCCACCAGTCG | Mouse |
| TF_qChIP_T6_R | TCCTGCAGTCTCTTCAGCTCAGCCTCACGC | Mouse |
| TF_qChIP_T7_F | ATTTCCCACTTCCAGTTCCAGCGTC | Mouse |
| TF_qChIP_T7_R | ACGCAGAACTCCCTGATGTCCACAGCATAC | Mouse |
| TF_qChIP_T8_F | TGTCTCCGAATTAGCAGGGACAGAGACTAT | Mouse |
| TF_qChIP_T8_R | CCCACCTGACTCAGCCATTAGTTCAGA | Mouse |
| TF_qChIP_T9_F | CAGGACTTCTGGCACACTCTGAGGGTTAGA | Mouse |
| TF_qChIP_T9_R | GGGGAAAGAATGTGGCTGCCTAGAC | Mouse |
| TF_qChIP_T10_F | GGCGAGTGCAGGAGGCGATAGGACGGGACT | Mouse |
| TF_qChIP_T10_R | CACCGCTCGCCACCAGGGGTCCGAA | Mouse |
| TF_qChIP_T11_F | AAGAGCAGAATAGTGGCAAAGCAAGTAGAT | Mouse |
| TF_qChIP_T11_R | TCTGTTCAATAGAAAGTTTCCCTAATCCTC | Mouse |
| TF_qChIP_T12_F | CTTGCCAGAAGATGATGAGATGAGGTATGC | Mouse |
| TF_qChIP_T12_R | CTCTTTCCCACTATGGTGCCTGCCTAAC | Mouse |
| TF_qChIP_T13_F | ATGAGTGGGAAGAGCCAGGAGATTTGCAAG | Mouse |
| TF_qChIP_T13_R | GAGTCATTTCCCGACAGGCTGTGTTGTAC | Mouse |
| TF_qChIP_T14_F | TTTTCAGAACAAGTCTCTCCCTAGCC | Mouse |
| TF_qChIP_T14_R | AACAGAGTCAGTTGAATGTTGAGTGCACCT | Mouse |
| TF_qChIP_T15_F | GAACAGATTTATACAATGTCCCGCGCATGT | Mouse |
| TF_qChIP_T15_R | TTATTCAGAAGCGGCTGCAGAAAGACGTCC | Mouse |
| TF_qChIP_T16_F | TGAGTGTGAGCTGGGCTAGTGTGGGCTAGT | Mouse |
| TF_qChIP_T16_R | CCAGAGCTCCCCCAGGACTGAGTCAT | Mouse |
| TF_qChIP_T17_F | CTCCACTTTGCCTTGGCTTACCGACAGTGC | Mouse |
| TF_qChIP_T17_R | GAGACTGAGAAAGGAGGGGCTTGAG | Mouse |
| TF_qChIP_T18_F | GACTGCCAGGAACAAACCATTACTAGCAAG | Mouse |

| | | |
|---|---|---|
| TF_qChIP_T18_R | GAAAAATGGCCACTGAAAACTGACACA | Mouse |
| TF_qChIP_T19_F | GGCAAGGGGGAACAAGTTTCTACGTT | Mouse |
| TF_qChIP_T19_R | GGATGGCCTCCTCCTTCCTTTTCTAATTTC | Mouse |
| TF_qChIP_T20_F | GCAGAAAGCCAGTTCAAAAGCCCTCTAGGA | Mouse |
| TF_qChIP_T20_R | GACACTATGGGCTGAATTTTCAATGACGC | Mouse |
| TF_qChIP_T21_F | AGAATAATGGCCGCTCTGAGACACCCAAG | Mouse |
| TF_qChIP_T21_R | TATCCAGGTGCCCCTAAACAGCAAGCTTTT | Mouse |
| TF_qChIP_T22_F | AGAGACATGCTGATTACATATCTAATTGGC | Mouse |
| TF_qChIP_T22_R | AAATAAGTAAACAAACAAACGCTCCC | Mouse |
| TF_qChIP_T23_F | GTCCTTACCAGCCCAGCGCCCCAAGCTTC | Mouse |
| TF_qChIP_T23_R | TTTTGCAGCAGTTGCGAAAACCCGGAACGA | Mouse |
| TF_qChIP_T24_F | CTCCAGAGAAAGAGACCTGACTCAGAACGG | Mouse |
| TF_qChIP_T24_R | CTCGCTCTTCCCCAGTGATAGGACTGT | Mouse |
| TF_qChIP_T25_F | GCCACTGGGACCTCCGCTGCGCTACTGTG | Mouse |
| TF_qChIP_T25_R | TGGGAGCGCAGCATCACTTCCTGTTGTGGG | Mouse |
| TF_qChIP_T26_F | CGATCGAGAAGGTGTTCCTAACCCCTAAAA | Mouse |
| TF_qChIP_T26_R | GCGGGAAAACAGCCAATATTATCAGC | Mouse |
| TF_qChIP_T27_F | GCTGCCTGCAACACATAAACACATACCAGT | Mouse |
| TF_qChIP_T27_R | TGCAAATCTACCTGGCATCTGACTAGAGAC | Mouse |
| TF_qChIP_T28_F | ACTACCCACTCAAAGACAGTCTGCGCATGT | Mouse |
| TF_qChIP_T28_R | ACTGTGGTGCATGAGCAATGGCGATAAGT | Mouse |
| TF_qChIP_T29_F | GCCACAACGCTCCAAATTCTCCAAGTCTGC | Mouse |
| TF_qChIP_T29_R | AGGGTCCTGGTGGGGTCTGGCCTATA | Mouse |
| TF_qChIP_T30_F | CTGGGGTGTAGGGAACCATTGTGCAGCGAT | Mouse |
| TF_qChIP_T30_R | GCGCTAGTACACACCAAGGCTCGAGGG | Mouse |
| TF_qChIP_T31_F | ACCTCCCACTGCGTATCCGGTGAATACCAC | Mouse |
| TF_qChIP_T31_R | GAAGACACTCATACACCCCTCCCATTAGGC | Mouse |
| TF_qChIP_T32_F | GGCTTCTAGAGCTGGCTGCTTGCTACA | Mouse |
| TF_qChIP_T32_R | GGAAAGTGCTCCAGGAAAACAAAGGCTATT | Mouse |
| TF_qChIP_T33_F | CAAAGTTTTAGGTTCAGCCTTCTAGTGG | Mouse |
| TF_qChIP_T33_R | TCGATGGCAGAGACTATCCTTTGATGACTG | Mouse |
| TF_qChIP_T34_F | AGTTCCAGGCAGGCGTTTCTAGCTTGCACC | Mouse |
| TF_qChIP_T34_R | ATTCACCAGTGAGCACCGGCCTTACC | Mouse |
| TF_qChIP_T35_F | GCCCTCTGAGTCCCAGCTTGATACATCC | Mouse |
| TF_qChIP_T35_R | TTCCTCCCCCTCTTTCCTTGTTCTCTAACT | Mouse |
| TF_qChIP_T36_F | CTTGTAACAGCAGCATTCCAGGATCTGATT | Mouse |
| TF_qChIP_T36_R | CCCATCATATCCCATTAAGCTAAGCC | Mouse |
| TF_qChIP_T37_F | CATATAACCTGGACGGAGCTGTTGACCTTG | Mouse |
| TF_qChIP_T37_R | GCTATGAAGGCCACCACAGAGTATGC | Mouse |

|  |  |  |
|---|---|---|
| TF_qChIP_T38_F | CAACTGGGACAGCAAGGACATTTATCGG | Mouse |
| TF_qChIP_T38_R | GTTGTAGCAGTCAGGGAAGCCATAGTAGCC | Mouse |
| TF_qChIP_T39_F | GCGGCAGCAGCTTCTCCTCCTCGGCGATAG | Mouse |
| TF_qChIP_T39_R | TTTCAGCACCTGAGGCCGCCGGACC | Mouse |
| TF_qChIP_T40_F | CCCCAACCATGTGACACGTGCAGCTAAGTC | Mouse |
| TF_qChIP_T40_R | GTGGCAGATCAGAGCACAAAGGGGTCAT | Mouse |
| TF_qChIP_T41_F | TTTTCCACCAGTGACCATGAAATCTAAAGT | Mouse |
| TF_qChIP_T41_R | GGAAATCCAGAGTGCACAGCTGACTACCT | Mouse |
| TF_qChIP_T42_F | GGATGGAAGCCACTCGTGCCTCTGCGTCA | Mouse |
| TF_qChIP_T42_R | CTGCCTGCTGTCCGGTTCTTTATCGAGCTA | Mouse |
| TF_qChIP_T43_F | GCCCTCAAGTAGAAGAGCTCAGTTAGAGT | Mouse |
| TF_qChIP_T43_R | AGACCGAAGCCTCTAAGTCTGTAGTACACA | Mouse |
| TF_qChIP_T44_F | TCTAAACCTCCCACCCAGCACAGTAGGCTC | Mouse |
| TF_qChIP_T44_R | CCTGATACTCCCCTGCCTCCCACTAAG | Mouse |
| TF_qChIP_T45_F | GACGCAGCCAGCCCAGGGAGAGTCGGATGC | Mouse |
| TF_qChIP_T45_R | TGGCTCGGGCCGCTGCACTTTCCAG | Mouse |
| TF_qChIP_T46_F | GAAGCTCCTCTGGTTGTGTAATCTGCGTCA | Mouse |
| TF_qChIP_T46_R | AAACAAACAGAACACGCAACTCGGTTTC | Mouse |
| TF_qChIP_T47_F | TCCCGACCATTGTTCTACTCTTCAGTCAGA | Mouse |
| TF_qChIP_T47_R | ATGGTTTGCTCCTTTTTAGCTAGTTCCC | Mouse |
| **Fig EV2_F** |  |  |
| **Name** | **Sequence** | **Species** |
| Oct4_qChIP_F | GGGCCTTCGTTCAGAGCAT | Mouse |
| Oct4_qChIP_R | TGCAAGAGAATAGCCCTCAGAGT | Mouse |
| Nanog_qChIP_F | ACACTAAAGAGGCAGGACAGGAA | Mouse |
| Nanog_qChIP_R | ACAGCTCCGGGTCAAAGGA | Mouse |
| Sox2_qChIP_F | ACAGTCGGTCGAAATGATGGA | Mouse |
| Sox2_qChIP_R | ACCAGGCCTAGCCTCTTCGT | Mouse |
| Esrrb_qChIP_F | GGTTGCTTCAAACAGAAGGAATG | Mouse |
| Esrrb_qChIP_R | GGGAGACTTATATGGGAGGGATTAA | Mouse |
| Prdm14_qChIP_F | GGTCAAATTTGTCAGAGAACCAGGTA | Mouse |
| Prdm14_qChIP_R | AAGCTGCAGAAAGGGCAAAA | Mouse |
| Klf4_qChIP_F | CCCCAAAGTCAACGAAGATTAAA | Mouse |
| Klf4_qChIP_R | AAGAAGAAGGATCTCGGGCAAT | Mouse |
| Rex1_qChIP_F | CCCGGACCCCGCTACA | Mouse |
| Rex1_qChIP_R | AGTGGTGAACGCTGCATGAC | Mouse |
| Rif1_qChIP_F | TTCTGCCGCACCTGTGTATC | Mouse |
| Rif1_qChIP_R | TGCCTAGAATTGGTTTACTTCCATT | Mouse |
| Bub1_qChIP_F | TATGTTCTGCTGGGTGATTG | Mouse |

| | | |
|---|---|---|
| Bub1_qChIP_R | GCTGAGAATGACCTTGAATTC | Mouse |
| Mcm10_qChIP_F | CTGAGCCTTTCTAGTCGGCCATAC | Mouse |
| Mcm10_qChIP_R | TGGTGGACAAATGTGCAGTTAAC | Mouse |
| Jmjd3_qChIP_F | GGAGATCGATGCCTATGACAAAG | Mouse |
| Jmjd3_qChIP_R | CATGTACTCCTGTCCCTGCTATTATTAT | Mouse |
| Cck_qChIP_F | TCTGCGTTTTACGGGAAGTCA | Mouse |
| Cck_qChIP_R | GCAGGGCTTTGGCCACAT | Mouse |
| Trak1_qChIP_F | TGTGTAGAATTCTGTGAGGAGTTGAGT | Mouse |
| Trak1_qChIP_R | GCATACATTGTCACAGCAGGAAA | Mouse |
| Ctnnb1_qChIP_F | GGAAGAGACGTGAGCTCAGAATAAT | Mouse |
| Ctnnb1_qChIP_R | CACGATTGGCACTTGTCAAAA | Mouse |

**Appendix Table 7. Primers used for gene expression analysis**

| **Fig.1 L** | | |
|---|---|---|
| **Name** | **Sequence** | **Species** |
| *Rif1_F* | GCAAGGATGTTGAGACTGAG | Mouse |
| *Rif1_R* | CAAGTATGTCCGTCTCCTTTAC | Mouse |
| *Bub1_F* | TGACCTGGGTCAGAGTATAG | Mouse |
| *Bub1_R* | AAGAGCCAAAGAGCATACAG | Mouse |
| *Mcm10_F* | AGTTCCAAACCTGCCATCCA | Mouse |
| *Mcm10_R* | TTCCGCATCTCCAGCATCTG | Mouse |
| *Trak1_F* | ACGAGCCCTCCCTACAACAAC | Mouse |
| *Trak1_R* | CGCACGGGCTCTTTTC | Mouse |
| *Ctnnb1_F* | ATGGAGCCGGACAGAAAAGC | Mouse |
| *Ctnnb1_R* | CTTGCCACTCAGGGAAGGA | Mouse |
| *Jmjd3_F* | TGAAGAACGTCAAGTCCATTGTG | Mouse |
| *Jmjd3_R* | TCCCGCTGTACCTGACAGT | Mouse |
| *Mobp_F* | AGTACAGCATCTGCAAGAGCG | Mouse |
| *Mobp_R* | TCCTCAATCTAGTCTTCTGGCA | Mouse |
| *Myo1e_F* | GGGTCACTCCACAAATGATCCTA | Mouse |
| *Myo1e_R* | CCCCGCTACTTACACCAGATTTA | Mouse |
| *Mycn_F* | ACAACATCCTGGAGCGTCAAC | Mouse |
| *Mycn_R* | GTTCTTCACCAGCTCAGGCACAT | Mouse |
| *Suv39h1_F* | CTGCAGGTGTACAACGTATTCATAGA | Mouse |
| *Suv39h1_R* | CAGATGGTTCTTGTGGCAAAGA | Mouse |
| *Tdrd6_F* | TGACGCCCAGGTGGAGTT | Mouse |
| *Tdrd6_R* | TCAAAATAACCCCATCCAGCTT | Mouse |
| **Fig.2 A** | | |

| Name | Sequence | Species |
|---|---|---|
| *Oct4_F* | ACATCGCCAATCAGCTTGG | Mouse |
| *Oct4_R* | AGAACCATACTCGAACCACATCC | Mouse |
| *Nanog_F* | TCTGGGAACGCCTCATCAAT | Mouse |
| *Nanog_R* | GGAGAGGCAGCCTCTGTGC | Mouse |
| *Dppa3_F* | CTTGTTCCGAGCTAGCTTTTGAG | Mouse |
| *Dppa3_R* | GGTCGACTTTCTCTGATGGTTCCT | Mouse |
| *Ncam1_F* | AGAAATCAGCGTTGGAGAGTCC | Mouse |
| *Ncam1_R* | TCGTCATCATTCCACACCACT | Mouse |
| *Myl2_F* | TCAGCTGCATTGACCAGAAC | Mouse |
| *Myl2_R* | CCCGAAGAGTGTGAGGAAGA | Mouse |
| *Gata4_F* | AGAGATGCGCCCCATCAAG | Mouse |
| *Gata4_R* | GCCGGACACAGTACTGAATGTC | Mouse |
| **Appendix Fig S3_G** | | |
| **Name** | **Sequence** | **Species** |
| *Cck_F* | CTGCTAGCGCGATACATCCA | Mouse |
| *Cck_R* | TCCAGGCTCTGCAGGTTCTT | Mouse |
| *Ttc21a_F* | GAGGCTCTCCGACTGAATGG | Mouse |
| *Ttc21a_R* | ACGTTGGCGACCGTGATG | Mouse |
| **Appendix Fig S3_H** | | |
| **Name** | **Sequence** | **Species** |
| *Cdk1_F* | AGAAGGTACTTACGGTGTGGT | Mouse |
| *Cdk1_R* | GAGAGATTTCCCGAATTGCAGT | Mouse |
| **Appendix Fig S4_A** | | |
| **Name** | **Sequence** | **Species** |
| *Sox2_F* | GCTCGCAGACCTACATGAACG | Mouse |
| *Sox2_R* | GCCTCGGACTTGACCACAGA | Mouse |
| *Gbx2_F* | GCAGTCGGTTGATTTTGGAG | Mouse |
| *Gbx2_R* | CGACATGGCTCAGATAGGAT | Mouse |
| *Dppa4_F* | TGAGGCCGTGTTAGCATCTTG | Mouse |
| *Dppa4_R* | CACACCACATTTCCCCTTTGA | Mouse |
| *Ddx4_F* | GGCAAAGAAAAGATTGGCCT | Mouse |
| *Ddx4_R* | GGGTTTGGCGTTGTTCCTT | Mouse |
| *Sycp3_F* | AGCCAGTAACCAGAAAATTGAGC | Mouse |
| *Sycp3_R* | CCACTGCTGCAACACATTCATA | Mouse |
| *Tfcp2l1_F* | GGCCTCCGTGAAGGTAACAG | Mouse |
| *Tfcp2l1_R* | GCTGATGGGAGCAGGTGATC | Mouse |
| **Appendix Fig S4_B** | | |
| **Name** | **Sequence** | **Species** |

| | | |
|---|---|---|
| *Bmp2_F* | GCATGTTTGGCCTGAAGCA | Mouse |
| *Bmp2_R* | GCCTGCGGTACAGATCTAGCA | Mouse |
| *Bmp4_F* | GGTGGGAAACTTTCGATGTGA | Mouse |
| *Bmp4_R* | CTCAATGGCCAGCCCATAAT | Mouse |
| *Bmp5_F* | GTGGGCTGGCTTGTCTTTGATA | Mouse |
| *Bmp5_R* | AGCTGTAAGCCCAAATTGTTCTG | Mouse |
| *Gata3_F* | GAGGTGGACGTACTTTTTAACATCGA | Mouse |
| *Gata3_R* | ACCGTAGCCCTGACGGAGTT | Mouse |
| *Gata6_F* | GCCGGAGGAAATGTACCAGAC | Mouse |
| *Gata6_R* | CCCCTTGAAGGTAGGGCAG | Mouse |
| *Sox17_F* | GATGCGGGATACGCCAGTG | Mouse |
| *Sox17_R* | CCACCACCTCGCCTTTCAC | Mouse |
| *Id1_F* | ACGACATGAACGGCTGCTACT | Mouse |
| *Id1_R* | TCTCCACCTTGCTCACTTTGC | Mouse |
| *Id2_F* | AGCATCCCCCAGAACAAGAA | Mouse |
| *Id2_R* | GCGATCTGCAGGTCCAAGAT | Mouse |
| *Id3_F* | TGGACGACATGAACCACTGCTA | Mouse |
| *Id3_R* | CTATGACACGCTGCAGGATTTC | Mouse |
| *Id4_F* | TGCGATATGAACGACTGCTACA | Mouse |
| *Id4_R* | TCCACTTTGCTGACTTTCTTGTTG | Mouse |
| *Foxa1_F* | GATGGAAGGGCATGAGAGCAA | Mouse |
| *Foxa1_R* | TGCTGACAGGGACAGAGGAGTA | Mouse |
| *Chd1_F* | GACGTCCATGTGTGTGACTG | Mouse |
| *Chd1_R* | CCTGCTGCCACGATTCCCGCC | Mouse |
| **Fig EV2_H** | | |
| **Name** | **Sequence** | **Species** |
| *Klf2_F* | AGGCCTGTGGGTTCGCTATAAA | Mouse |
| *Klf2_R* | GGCAAATTATGGCTCAAAGTAGCAG | Mouse |
| *Klf4_F* | CCAGACCAGATGCAGTCACAA | Mouse |
| *Klf4_R* | TGGCATGAGCTCTTGATAATGG | Mouse |
| *Esrrb_F* | TGGGCCTAGCAGGGTCAGA | Mouse |
| *Esrrb_R* | TGCCACCTGTTTCTCATGAGTAG | Mouse |
| *Prdm14_F* | CAATTTCAGGCATCCTGGTTC | Mouse |
| *Prdm14_R* | GTGGCACATCACCAAATGAGG | Mouse |
| *cMyc_F* | ACCAGCAGCGACTCTGAAGAAG | Mouse |
| *cMyc_R* | GTTTGCCTCTTCTCCACAGACA | Mouse |
| **Fig EV2_I** | | |
| **Name** | **Sequence** | **Species** |
| *Lef1_F* | CACCTACAGCGACGAGCACTT | Mouse |

| | | |
|---|---|---|
| *Lef1_R* | ACATGCCTTGCTTGGAGTTGA | Mouse |
| *Nestin_F* | CCCTGAAGTCGAGGAGCTG | Mouse |
| *Nestin_R* | CTGCTGCACCTCTAAGCGA | Mouse |
| *Gdpd5_F* | TACAACCGCATGGGCTACTG | Mouse |
| *Gdpd5_R* | CCTGCGATGTATGTGAAAGCA | Mouse |
| *Xirp1_F* | CGATGTGAAGAAGACGGTGAAG | Mouse |
| *Xirp1_R* | ACCTCACTGCATTGCTTTGAATC | Mouse |
| **Fig EV5_C** | | |
| **Name** | **Sequence** | **Species** |
| *CDK1_F* | AAACTACAGGTCAAGTGGTAGCC | Human |
| *CDK1_R* | TCCTGCATAAGCACATCCTGA | Human |
| **Fig EV5_J** | | |
| **Name** | **Sequence** | **Species** |
| *RIF1_F* | TGTGCTGAGCTTAGAGCCATTG | Human |
| *RIF1_R* | TGATAAGTGTATTTGCATGTTTGGAA | Human |
| *BUB1_F* | GGCAGAGTTGGGCGTTGAG | Human |
| *BUB1_R* | TGCTTGGAGCCCAGCAATA | Human |
| *MCM10_F* | CAAGAGGGTGGCTCGAACA | Human |
| *MCM10_R* | GGGAGGGTGCACTTGTCATC | Human |
| *TRAK1_F* | CACCAGCCACTTGAAATCCA | Human |
| *TRAK1_R* | CACGGGTGTTAGTGAAGGACTCA | Human |
| *JMJD3_F* | CCCCTCACCGCCTATCAGTA | Human |
| *JMJD3_R* | CCGTGCGAGCCACGTT | Human |
| *CD44_F* | CTGCCGCTTTGCAGGTGTA | Human |
| *CD44_R* | CATTGTGGGCAAGGTGCTATT | Human |
| *STAT3_F* | GAGAAGGACATCAGCGGTAAGAC | Human |
| *STAT3_R* | AGACCAGTGGAGACACCAGGAT | Human |
| *CTNNB1_F* | CATCTACACAGTTTGATGCTGCT | Human |
| *CTNNB1_R* | GCAGTTTTGTCAGTTCAGGGA | Human |
| *ERAS_F* | GGACCACGACCCCACCAT | Human |
| *ERAS_R* | GCCCAGCACACCATCACA | Human |
| *BMP2_F* | AAAAACGTCAAGCCAAACACAA | Human |
| *BMP2_R* | GTCACTGAAGTCCACGTACAAAGG | Human |
| *GATA4_F* | TGTCCTCTCGGCCCTGAA | Human |
| *GATA4_R* | TCCTGCTTGGAGCTGGTCTGT | Human |
| *GATA6_F* | GATTGTCCTGTGCCAACTGTCA | Human |
| *GATA6_R* | AGCATTGCACACGGGTTCAC | Human |
| *ID1_F* | ACGTGCTGCTCTACGACATGA | Human |
| *ID1_R* | TGCTCACCTTGCGGTTCTG | Human |

| | | |
|---|---|---|
| *ID2_F* | CAGCATCCCCCAGAACAAGAA | Human |
| *ID2_R* | CGATCTGCAGGTCCAAGATGT | Human |
| *LEF1_F* | GGCCAGACAAGCACAAACCT | Human |
| *LEF1_R* | CATCTGGATGCTTTCCGTCAT | Human |

**Appendix Table 8. Primers used for ORF cloning and site-directed mutagenesis**

| | |
|---|---|
| **Murine Tfcp2l1 ORF** | |
| mTfcp2l1_ORF_F | GGATCCGCCACCATG-CTGTTCTGGCACACGCAG |
| mTfcp2l1_ORF_R | CTCGAGTCAGAGTCCACACTTCAGGAT |
| mTfcp2l1-T177A_S (sense) | GTATCAGCACGGAATTC**G**CCCCCAGGAAGCA |
| mTfcp2l1-T177A_AS (anti-sense) | TGCTTCCTGGGGG**C**GAATTCCGTGCTGATAC |
| mTfcp2l1-T177E_S (sense) | CTGTATCAGCACGGAATTC**GAA**CCCAGGAAGCATGGGGG |
| mTfcp2l1-T177E_AS (anti-sense) | CCCCCATGCTTCCTGGG**TTC**GAATTCCGTGCTGATACAG |
| **Human TFCP2L1 ORF** | |
| hTFCP2L1-T177A_S (sense) | TGCATCAGCACAGAATTC**G**CCCCCAGGAAGCA |
| hTFCP2L1-T177A_AS (anti-sense) | TGCTTCCTGGGGG**C**GAATTCTGTGCTGATGCA |
| hTFCP2L1-T177E_S (sense) | TGCATCAGCACAGAATTC**GAG**CCCAGGAAGCACGGGG |
| hTFCP2L1-T177E_AS (anti-sense) | CCCCGTGCTTCCTGGG**CTC**GAATTCTGTGCTGATGCA |

**Appendix Table 9. Information on target sequences in shRNA**

| | |
|---|---|
| LacZ | GCTACACAAATCAGCGATT |
| Murine *Tfcp2l1* (#1) | GCAGGAATGTGAGGCCAAAGA |
| Murine *Tfcp2l1* (#2) | GCTCTTCAATGCCATCAAAGG |
| Murine *Cdk1* (#1) | TACTTACGGTGTGGTGTATAA |
| Murine *Cdk1* (#2) | GCCAGATAGTGGCCATGAAGA |
| Murine *Cdk1* (#3) | GGACTACAAGAACACCTTTCC |

| | |
|---|---|
| Murine *Cdk1* (#4) | GCTGTATCTCATCTTTGAGTT |
| Murine *Cdk1* (#5) | GCCAAACGAATCTCTGGCAAA |
| Human *TFCP2L1* (#1) | GCTCTTCAACGCCATCAAAGG |
| Human *TFCP2L1* (#2) | CGAGTCCAGATTGACACGTTT |
| Human *CDK1* (#1) | GTGGAATCTTTACAGGACTAT |
| Human *CDK1* (#2) | GCTGTACTTCGTCTTCTAATT |
| Human *CDK1* (#3) | TGGCTTGGATTTGCTCTCGAA |
| Human *CDK1* (#4) | GTTTCCATATGTTATGTCAAC |
| Human *CDK1* (#5) | GATTCAGAAATTGATCAACTC |
