# Supplementary material for: Phosphorylation of TFCP2L1 by CDK1 is required for stem cell pluripotency and bladder carcinogenesis
Source: EMBO Mol Med. 2019 Nov 11;12(1):e10880. doi: 10.15252/emmm.201910880 (PMC6949511; doi:10.15252/emmm.201910880)
Supplement: Supplementary file 2 — Expanded View Figures PDF [file EMMM-12-e10880-s002.pdf]

Expanded View Figures

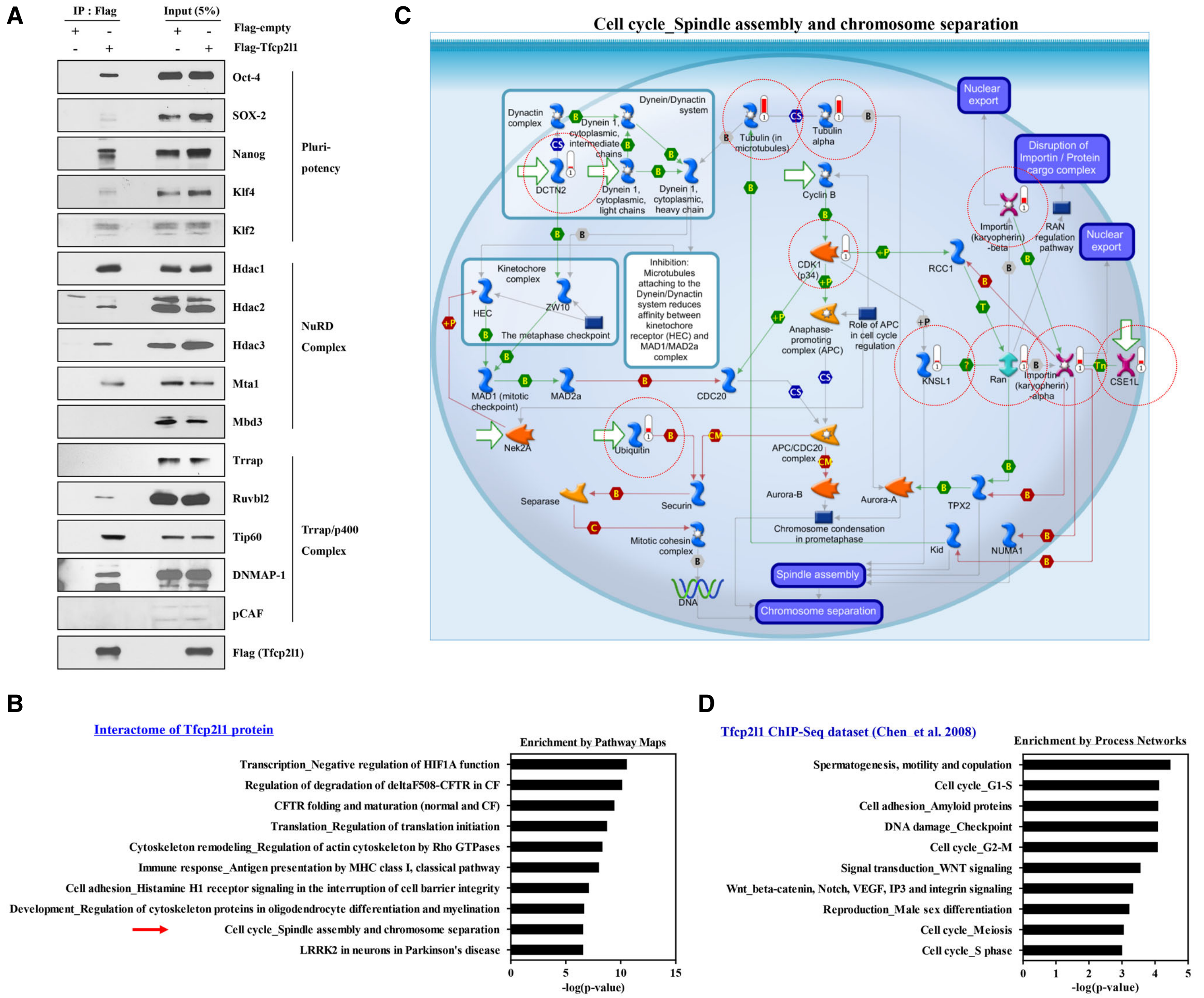

Figure EV1. Molecular features of Tfcp2l1 interactome and transcription targets in mESCs.

A Proteins in anti-FLAG immunoprecipitates (IP) of extracts of mESCs expressing Flag-Tfcp2l1 or Flag only. Co-precipitated proteins were identified with specific antibodies. Input (5%) shows proteins present in 5% of the input used for immunoprecipitation.

B, C The 10 most highly enriched Pathway Maps (B) and a schematic overview (C) of spindle assembly and chromosome separation-related cell cycle pathway (red-colored arrow in B) in MetaCore analysis of the Tfcp2l1 interactome dataset (Dataset EV1).

D The 10 most highly enriched Pathway Maps uncovered by MetaCore analysis of the Tfcp2l1 ChIP-seq database (Dataset EV2).

Source data are available online for this figure.

© 2019 The Authors

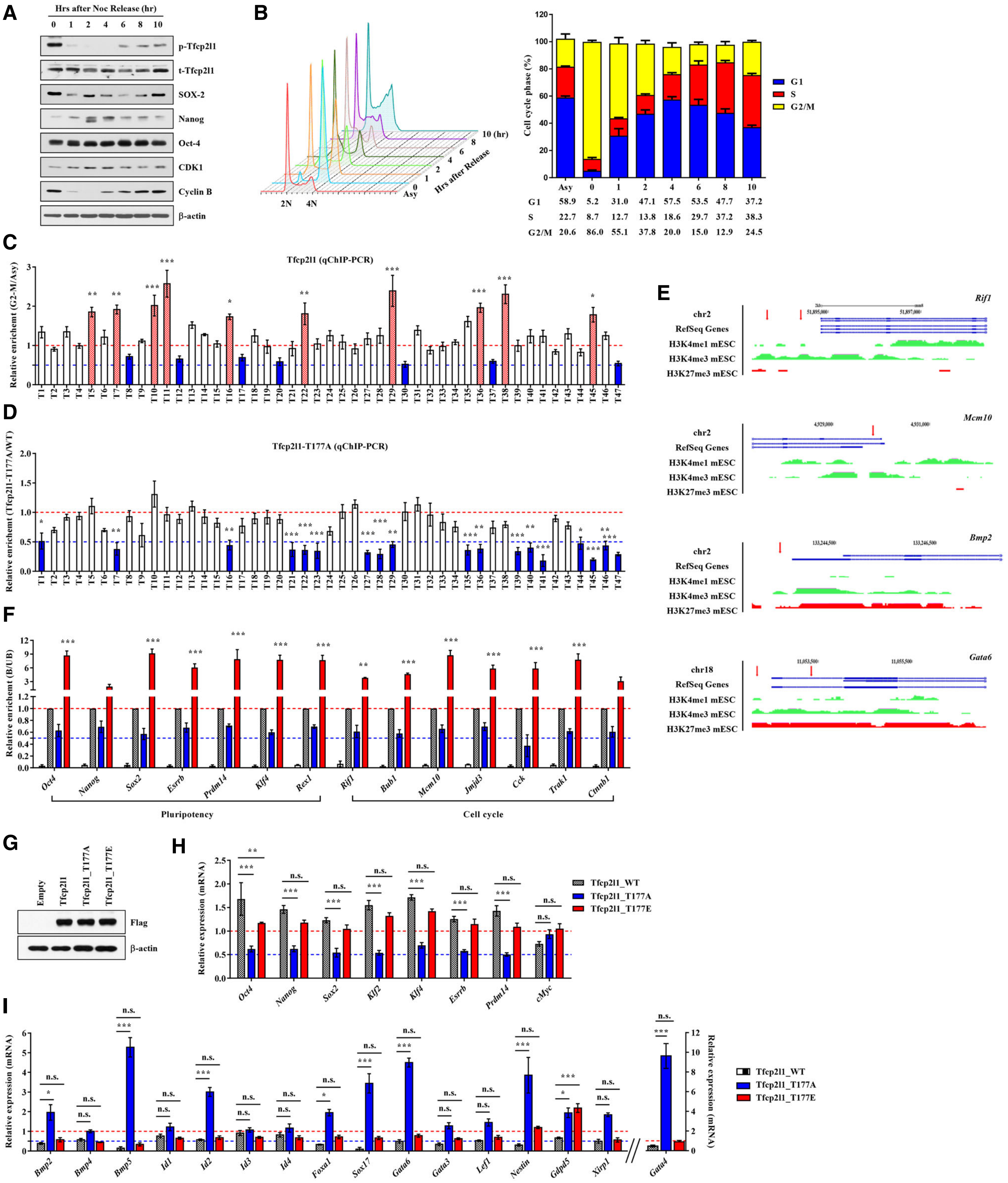
A
Hrs after Noc Release (hr)
0 1 2 4 6 8 10
p-Tfcp2l1
t-Tfcp2l1
SOX-2
Nanog
Oct-4
CDK1
Cyclin B
β-actin
B
2N 4N
Asy 0 1 2 4 6 8 10 (hr)
Hrs after Release
Cell cycle phase (%)
G1
S
G2/M
Asy 0 1 2 4 6 8 10
G1 58.9 5.2 31.0 47.1 57.5 53.5 47.7 37.2
S 22.7 8.7 12.7 13.8 18.6 29.7 37.2 38.3
G2/M 20.6 86.0 55.1 37.8 20.0 15.0 12.9 24.5
C
Tfcp2l1 (qChIP-PCR)
Relative enrichment (G2-M/Asy)
D
Tfcp2l1-T177A (qChIP-PCR)
Relative enrichment (Tfcp2l1-T177A/WT)
E
Rif1
Mcm10
Bmp2
Gata6
chr2
chr18
RefSeq Genes
H3K4me1 mESC
H3K4me3 mESC
H3K27me3 mESC
F
Relative enrichment (B/UB)
Oct4 Nanog Sox2 Esrrb Prdm14 Klf4 Rex1 Rif1 Bub1 Mcm10 Jmjd3 Cck Trak1 Ctnnb1
Pluripotency
Cell cycle
G
Empty
Tfcp2l1
Tfcp2l1_T177A
Tfcp2l1_T177E
Flag
β-actin
H
Relative expression (mRNA)
Tfcp2l1_WT
Tfcp2l1_T177A
Tfcp2l1_T177E
Oct4 Nanog Sox2 Klf2 Klf4 Esrrb Prdm14 cMyc
I
Relative expression (mRNA)
Bmp2 Bmp4 Bmp5 Id1 Id2 Id3 Id4 Foxa1 Sox17 Gata6 Gata3 Lef1 Nestin Gdpd5 Xirp1 Gata4
Tfcp2l1_WT
Tfcp2l1_T177A
Tfcp2l1_T177E

Figure EV2.

Figure EV2. Cell cycle-dependent Tfcp2l1 Thr177 phosphorylation determines the transcription targets by direct binding.

A Western blot analysis of proteins in mESCs in which the cell cycle was arrested in G2/M phase by treatment with nocodazole (Noc, 200 ng/ml) for 10 h followed by release. In a previous study, this condition arrested mESCs at G2/M phase without inducing differentiation (Shin *et al*, 2016). The polyclonal antibody for TFCP2L1 phosphorylated at Thr177 used for immunostaining of tumor tissues from bladder cancer patients was also reactive to Thr177 phosphorylation of the murine Tfcp2l1 protein (Appendix Fig S2).

B FACS analysis of propidium iodide stained cells and histograms showing the proportion of cells in each cell cycle phase after release of nocodazole-synchronized cells (G2/M phase) into normal medium.

C qChIP analysis of Flag-Tfcp2l1 binding to validated transcription targets (T1–T47) in G2/M-arrested and asynchronized (Asy) Flag-Tfcp2l1 mESCs. Fold difference is the ratio of G2/M-arrested to Asy mESCs (set to 1, red dotted line). Blue lines indicate half of the values of control groups. Binding of Tfcp2l1 to target chromatin in G2/M cells was higher than in asynchronized cells in some regions and lower in others.

D qChIP analysis in mESCs transiently transfected with Flag-Tfcp2l1-T177A. Notably, the majority of sites with upregulation of binding in G2/M phase also showed lower levels of binding by Tfcp2l1-T177A than by wild-type Tfcp2l1.

E Representative Tfcp2l1-targeted (*Rif1*, *Mcm10*, *Bmp2*, and *Gata6*) gene loci marked with regions enriched with H3K4me1, H3K4me3, and H3K27me3 in mESCs. Red arrows; the location of primer sets used in the ChIP assay. Genes located within 10 kb upstream or downstream from the affected Tfcp2l1 target sites were characterized by the Gene Ontology (GO) terms of cell cycle, proliferation, and differentiation processes (Appendix Table S1).

F qChIP analysis of Tfcp2l1 target genes involved in the cell cycle. Data are expressed as ratios relative to the values in Flag-Tfcp2l1-WT samples.

G Western blot analysis of Flag-tagged Tfcp2l1-WT, T177A, and T177E proteins in mESCs used in qChIP assay, confirming that these proteins were present at similar levels.

H, I Real-time qPCR analysis of Tfcp2l1 transcription targets related to pluripotency (H) and differentiation (I) in mESCs infected with *Tfcp2l1*-WT, T177A, and T177E-expressing lentiviruses. Lentivirus with no inserted coding sequence was used as the control.

Data information: All quantitative data are represented as means ± SEM. *$P < 0.05$, **$P < 0.01$, ***$P < 0.001$; n.s. = non-significant; two-way ANOVA with Bonferroni *post hoc* tests. Number of biological replicates is $n \geq 3$. The exact *P*-values and number of replicates are indicated in source data.

Source data are available online for this figure.

© 2019 The Authors

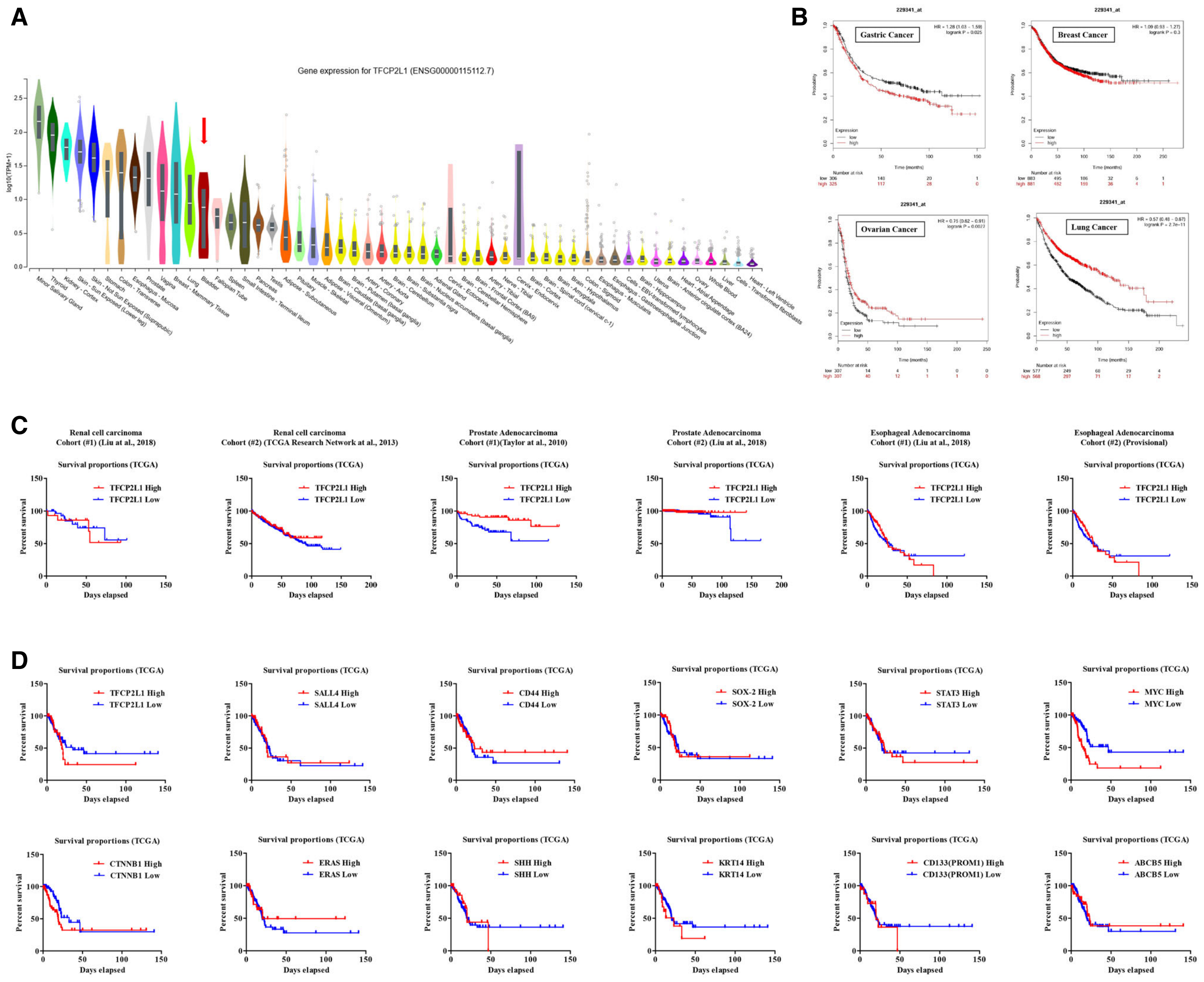

Figure EV3. Clinical significance of TFCP2L1 and stem cell markers in survival of cancer patients.

A TFCP2L1 (ENSG00000115112.7) gene expression in adult tissues obtained from Genotype-Tissue Expression (GTEx) datasets (https://gtexportal.org/home/). Expression values are shown in log scale of transcripts per million (TPM), and plots are shown as median and 25th and 75th percentiles. Data source: GTEx Analysis Release V7 (dbGaP Accession phs000424.v7.p2).

B Survival of gastric (n = 631), breast (n = 1,764), ovarian (n = 614), and lung (n = 1,145) cancer patients according to high (red) and low (black) expression levels of *TFCP2L1* using KM plotter (http://kmplot.com) databases.

C Survival of renal cell carcinoma (The Cancer Genome Atlas Research *et al*, 2013; Liu *et al*, 2018), prostate adenocarcinoma (Taylor *et al*, 2010; Liu *et al*, 2018), and esophageal adenocarcinoma (Liu *et al*, 2018) patients according to high (red) and low (black) expression levels of *TFCP2L1* in two independent cohorts from the TCGA datasets.

D Clinical significance of TFCP2L1 and cancer stem cell markers in bladder cancers. Kaplan–Meier plots of overall survival in patients with high or low expression levels of the indicated cancer stem cell markers in a bladder cancer cohort from the TCGA datasets.

Data information: The number of analyzed patients in (C and D) are indicated in source data.

Source data are available online for this figure.

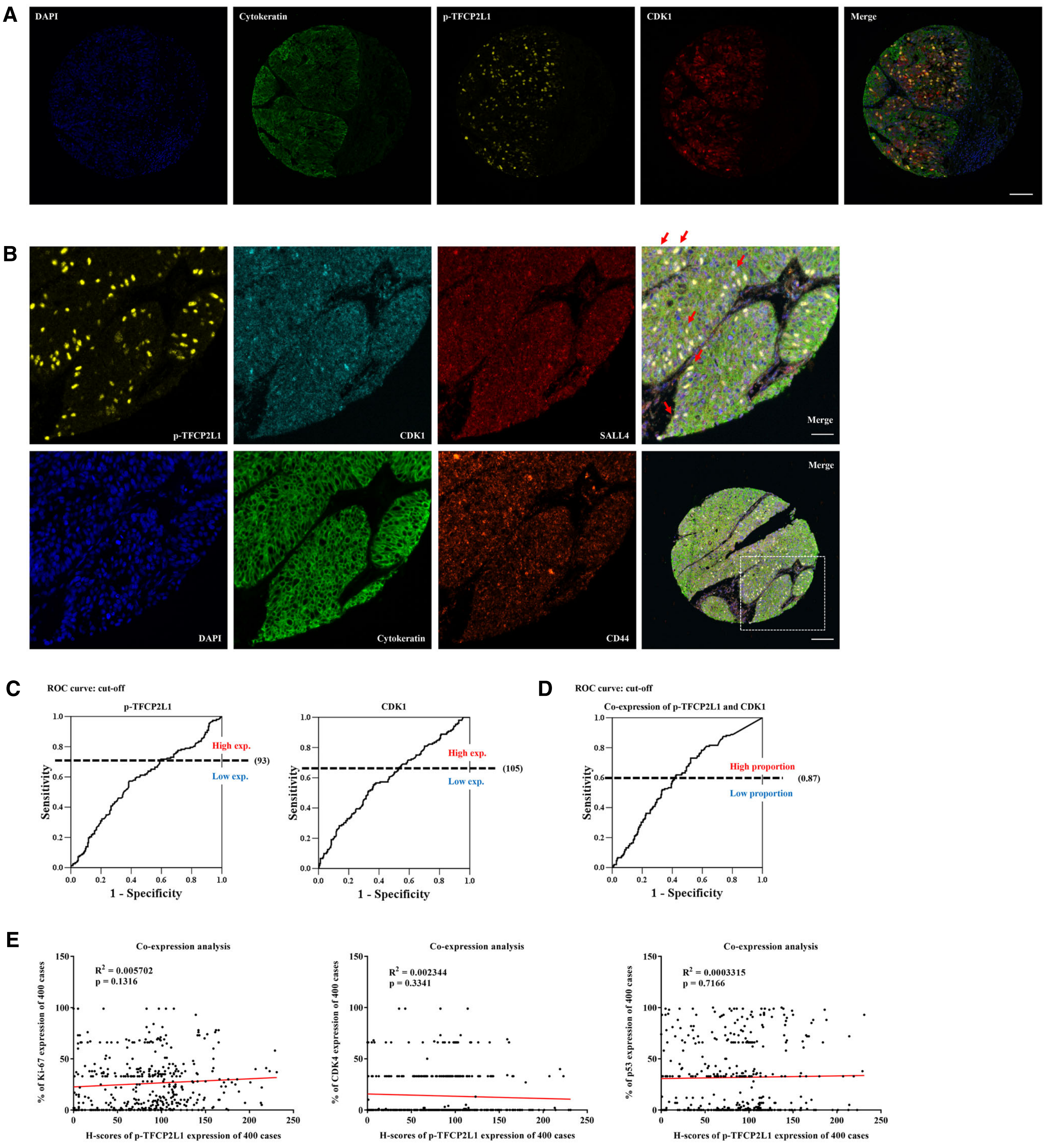

Figure EV4. Co-expression of p-TFCP2L1 and CDK1 with stem cell marker proteins in aggressive bladder cancers.

A, B Opal multiplex immunofluorescence staining of p-TFCP2L1 and CDK1 (A) and p-TFCP2L1, CDK1, SALL4, and CD44 stem cell markers (B) at ×200 magnification (scale bar = 100 μm) or at ×400 magnification (scale bar = 50 μm). Red arrows indicate the p-TFCP2L1$^+$ cells co-expressing CDK1 and SALL4.

C, D ROC analysis to determine cutoffs of high versus low expression of p-TFCP2L1 or CDK1 (C) or co-expression of p-TFCP2L1 and CDK1 proteins (D) in a tissue microarray from transurethral resection specimens of bladder tumors from 400 patients at our institute. ROC: Receiver operating characteristic curve.

E Co-expression analysis of p-TFCP2L1 with Ki-67, CDK4, and p53 proteins based on their H-scores.

Source data are available online for this figure.

© 2019 The Authors

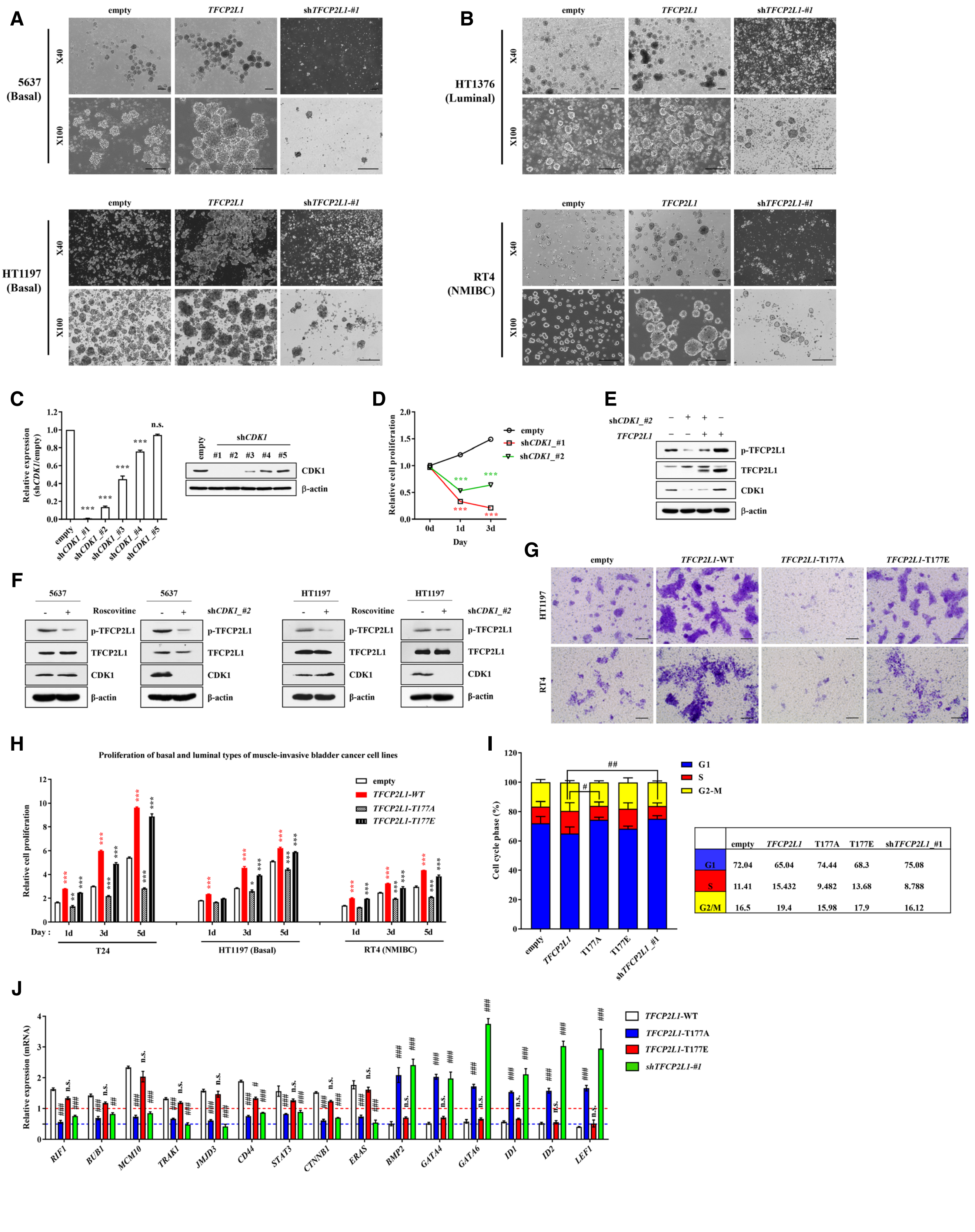

	empty	TFCP2L1	T177A	T177E	shTFCP2L1_#1
G1	72.04	65.04	74.44	68.3	75.08
S	11.41	15.432	9.482	13.68	8.788
G2/M	16.5	19.4	15.98	17.9	16.12

Figure EV5.

Figure EV5. Role of TFCP2L1 Thr177 phosphorylation on tumorigenic potency in basal and luminal subtypes of bladder cancer cells.

A, B Tumor sphere formation and cell proliferation assays using basal (A) and luminal (B) subtypes of BC cell lines with ectopic expression or silencing of *TFCP2L1*. Representative images from the tumor sphere formation assay are shown at ×40 (upper panel) or ×100 (lower panel) magnification. Scale bars = 200 μm. RT4 cell line represents a model of high-grade non-muscle-invasive BC (NMIBC).

C Western blot (right panel) and real-time qPCR (left panel) results of *CDK1*-silenced (sh*CDK1*) T24 cells. Five independent sh*CDK1* constructs were used.

D Cell viability of T24 cells carrying the indicated sh*CDK1* constructs, as determined by the MTT assay on the indicated days.

E Western blot results of CDK1 and total or T177 phosphorylated TFCP2L1 (p-TFCP2L1) proteins in *CDK1*-silenced T24 cells in the absence or presence of TFCP2L1 rescue.

F Western blot results of basal subtypes of BC cell lines following treatment with 25 μM roscovitine for 5 h (to inhibit CDK1) and basal subtypes BC cell lines with transient expression of shRNA for *CDK1* (sh*CDK1*). Note that interference with the activity or expression of CDK1 reduced the level of p-TFCP2L1 protein.

G Matrigel invasion assays in HT1197 basal type and RT4 luminal type BC cell lines infected with lentiviruses containing *TFCP2L1*-WT (wild-type), T177A, or T177E constructs. Representative images are shown at ×200 magnification. Scale bars = 100 μm.

H Cell proliferation assay in the indicated BC cell lines.

I, J Proportions of cells in each cell cycle phase (I) and expression of cell cycle, stemness, and differentiation related genes (J) in T24 cells carrying empty, *TFCP2L1*-WT, T177A, or T177E constructs.

Data information: All quantitative data are represented as means ± SEM. $^*P < 0.05$, $^{**}P < 0.01$, $^{***}P < 0.001$ compared with empty control. $^{\#}P < 0.05$, $^{\#\#}P < 0.01$, $^{\#\#\#}P < 0.001$ compared with *TFCP2L1*-WT, n.s. = not significant. Statistical tests used are as follows: one-way (C) and two-way ANOVA (D, H, I, and J) with Bonferroni *post hoc* tests. Number of biological replicates is $n \geq 4$. The exact *P*-values and number of replicates are indicated in source data.

Source data are available online for this figure.

 © 2019 The Authors
