# Supplementary material for: Phosphorylation of TFCP2L1 by CDK1 is required for stem cell pluripotency and bladder carcinogenesis
Source: EMBO Mol Med. 2019 Nov 11;12(1):e10880. doi: 10.15252/emmm.201910880 (PMC6949511; doi:10.15252/emmm.201910880)
Supplement: Supplementary file 5 — Source Data for Expanded View and Appendix [file EMMM-12-e10880-s012.zip › Heoetal_Source_data_EV_Appendix/Heoetal_Source_data_uncropped_Appendix_Fig_S3.pdf]

Appendix Fig S3

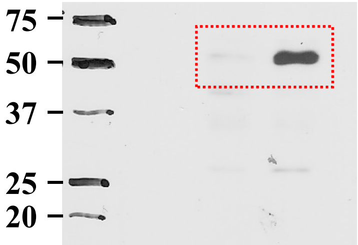

Appendix Fig S3B
(Tfcp2l1 WB)

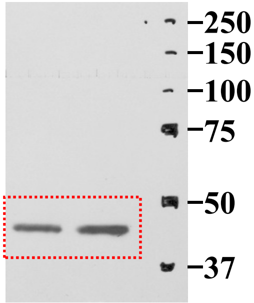

Appendix Fig S3B
(Oct-4 WB)

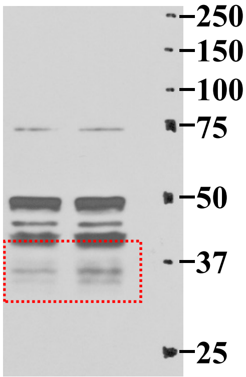

Appendix Fig S3B
(Nanog WB)

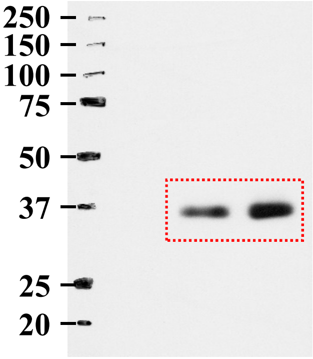

Appendix Fig S3B
(SOX-2 WB)

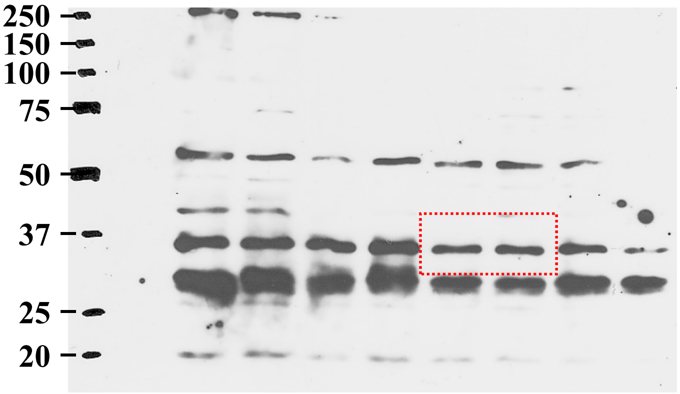

Appendix Fig S3B
(Cyclin D WB)

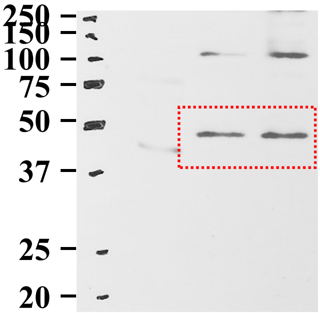

Appendix Fig S3B
(Cyclin E WB)

Appendix Fig S3

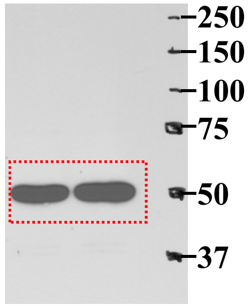

Appendix Fig S3B
(Cyclin A WB)

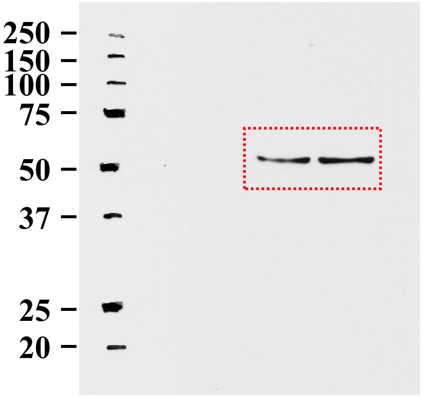

Appendix Fig S3B
(Cyclin B WB)

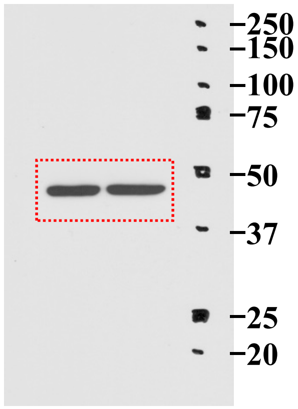

Appendix Fig S3B
(β-actin WB)

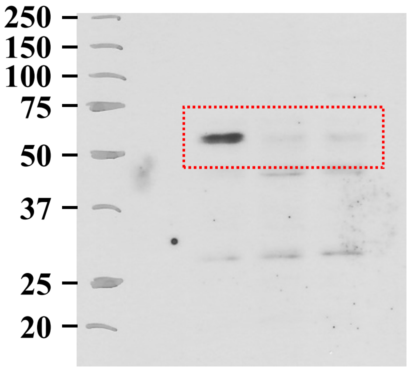

Appendix Fig S3E
(Tfcp2l1 WB)

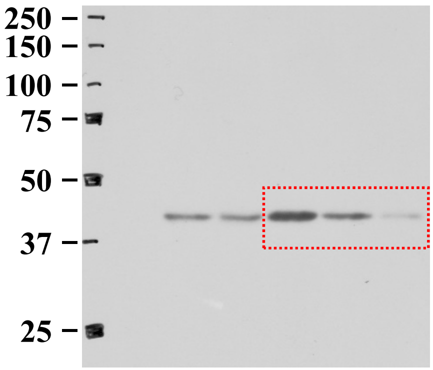

Appendix Fig S3E
(Oct-4 WB)

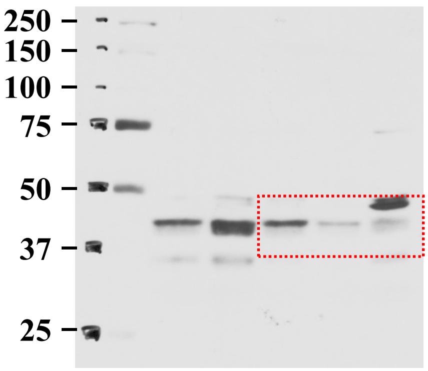

Appendix Fig S3E
(Nanog WB)

Appendix Fig S3

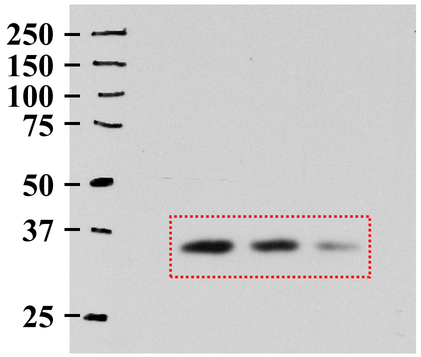

Appendix Fig S3E
(SOX-2 WB)

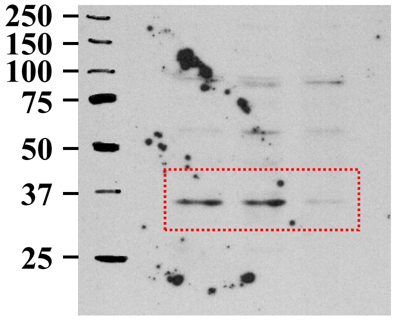

Appendix Fig S3E
(Cyclin D WB)

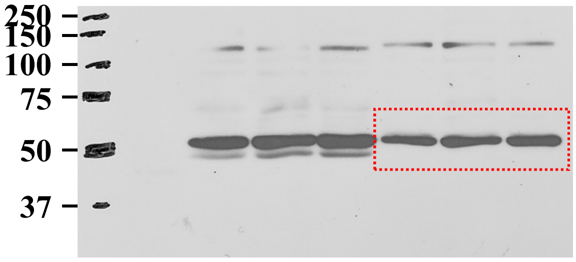

Appendix Fig S3E
(Cyclin E WB)

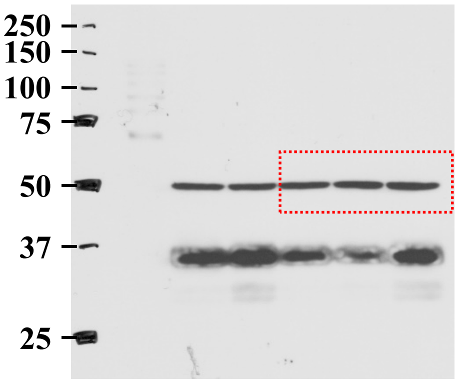

Appendix Fig S3E
(Cyclin A WB)

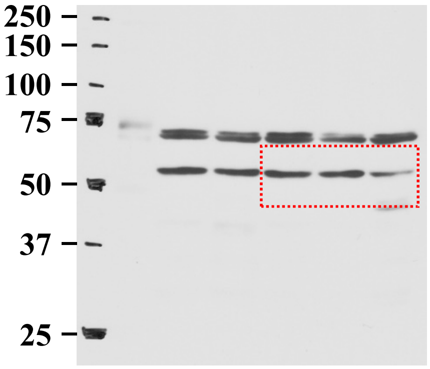

Appendix Fig S3E
(Cyclin B WB)

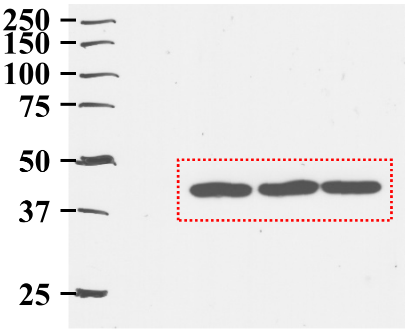

Appendix Fig S3E
(β-actin WB)

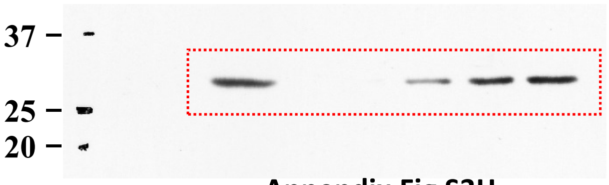

Appendix Fig S3H
(Cdk1 WB)
(left panel)

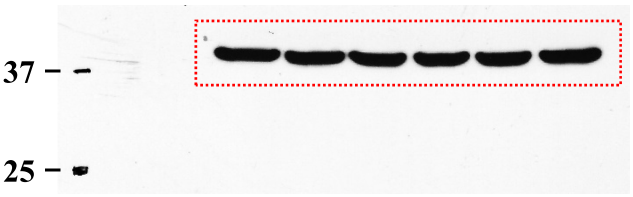

Appendix Fig S3H
(β-actin WB)
(left panel)
