# Supplementary material for: Phosphorylation of TFCP2L1 by CDK1 is required for stem cell pluripotency and bladder carcinogenesis
Source: EMBO Mol Med. 2019 Nov 11;12(1):e10880. doi: 10.15252/emmm.201910880 (PMC6949511; doi:10.15252/emmm.201910880)
Supplement: Supplementary file 5 — Source Data for Expanded View and Appendix [file EMMM-12-e10880-s012.zip › Heoetal_Source_data_EV_Appendix/Heoetal_Source_data_uncropped_Fig_EV5.pdf]

Fig EV5

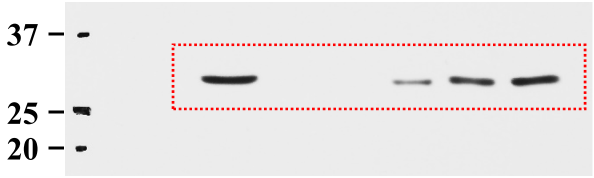

Fig EV5C
(CDK1 WB)
(right panel)

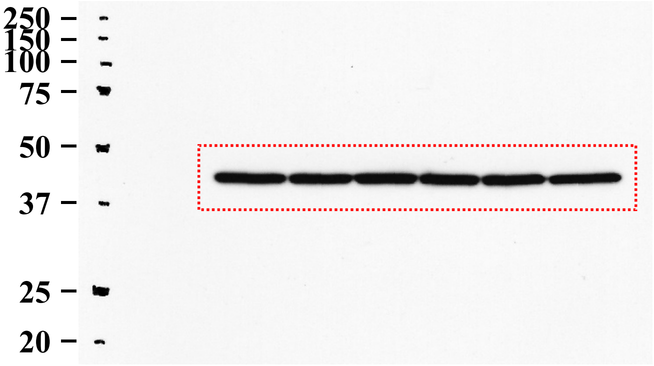

Fig EV5C
(β-actin WB)
(right panel)

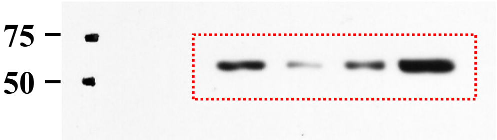

Fig EV5E
(p-TFCP2L1 WB)

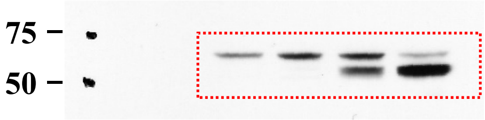

Fig EV5E
(TFCP2L1 WB)

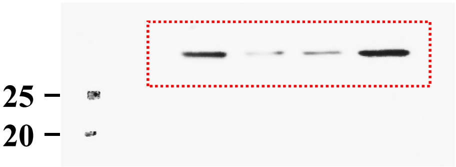

Fig EV5E
(CDK1 WB)

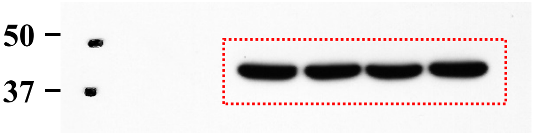

Fig EV5E
(β-actin WB)

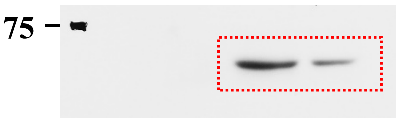

Fig EV5F
(p-TFCP2L1 WB)
(5637, Roscovitine)

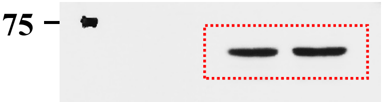

Fig EV5F
(t-TFCP2L1 WB)
(5637, Roscovitine)

Fig EV5

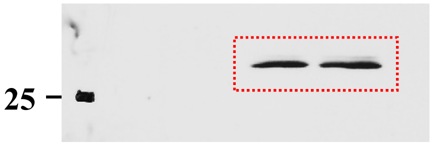

Fig EV5F
(CDK1 WB)
(5637, Roscovitine)

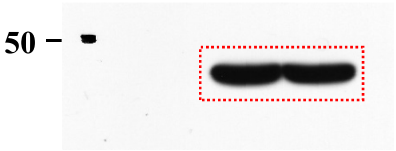

Fig EV5F
(β-actin WB)
(5637, Roscovitine)

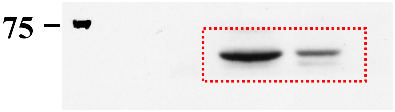

Fig EV5F
(p-TFCP2L1 WB)
(5637, shCDK1)

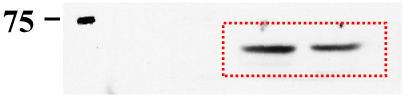

Fig EV5F
(t-TFCP2L1 WB)
(5637, shCDK1)

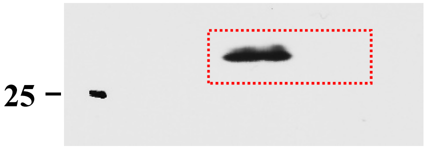

Fig EV5F
(CDK1 WB)
(5637, shCDK1)

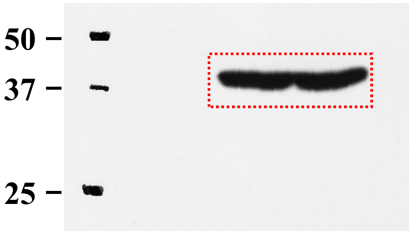

Fig EV5F
(β-actin WB)
(5637, shCDK1)

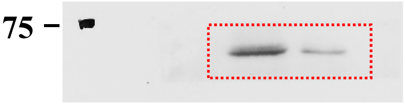

Fig EV5F
(p-TFCP2L1 WB)
(HT1197, Roscovitine)

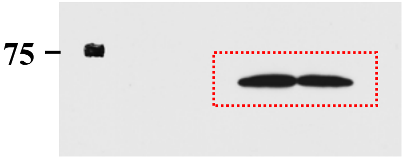

Fig EV5F
(t-TFCP2L1 WB)
(HT1197, Roscovitine)

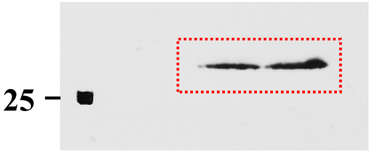

Fig EV5F
(CDK1 WB)
(HT1197, Roscovitine)

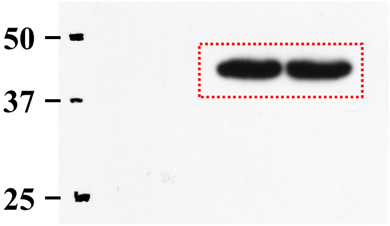

Fig EV5F
(β-actin WB)
(HT1197, Roscovitine)

Fig EV5

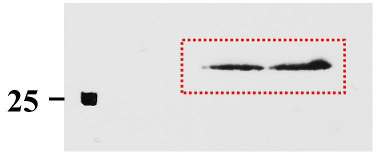

Fig EV5F
(CDK1 WB)
(HT1197, Roscovitine)

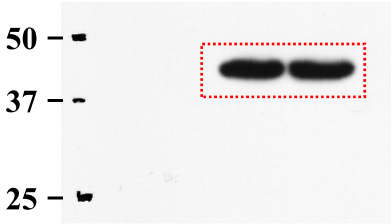

Fig EV5F
(β-actin WB)
(HT1197, Roscovitine)

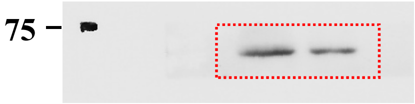

Fig EV5F
(p-TFCP2L1 WB)
(HT1197, shCDK1)

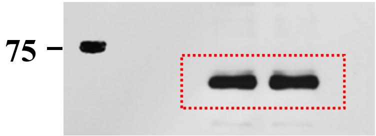

Fig EV5F
(t-TFCP2L1 WB)
(HT1197, shCDK1)

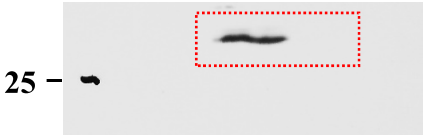

Fig EV5F
(CDK1 WB)
(HT1197, shCDK1)

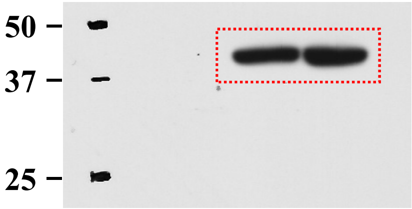

Fig EV5F
(β-actin WB)
(HT1197, shCDK1)
