# Supplementary material for: Phosphorylation of TFCP2L1 by CDK1 is required for stem cell pluripotency and bladder carcinogenesis
Source: EMBO Mol Med. 2019 Nov 11;12(1):e10880. doi: 10.15252/emmm.201910880 (PMC6949511; doi:10.15252/emmm.201910880)
Supplement: Supplementary file 5 — Source Data for Expanded View and Appendix [file EMMM-12-e10880-s012.zip › Heoetal_Source_data_EV_Appendix/Heoetal_Source_data_uncropped_Appendix_Fig_S2.pdf]

Appendix Fig S2

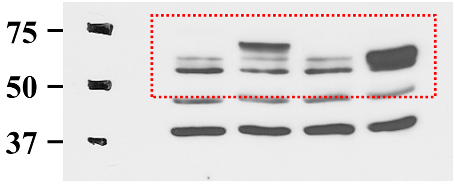

Appendix Fig S2B
(p-Tfcp2l1 (w/o peptide_WB)

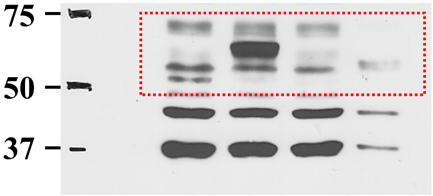

Appendix Fig S2B
(p-Tfcp2l1 (non-phospho-peptide_WB)

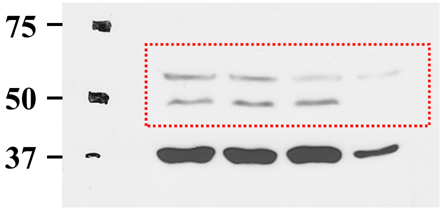

Appendix Fig S2B
(p-Tfcp2l1 (phospho-peptide_WB)

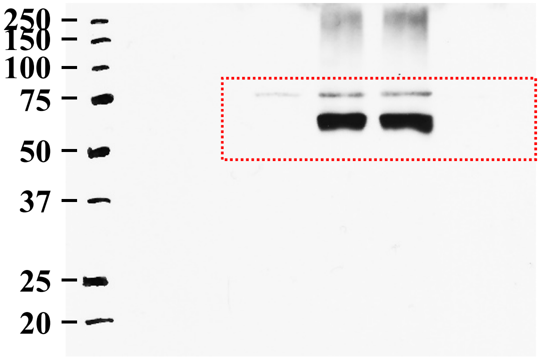

Appendix Fig S2B
(Flag WB)

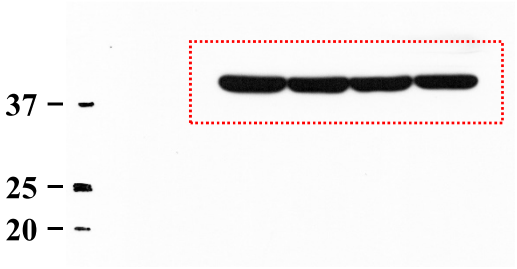

Appendix Fig S2B
(β-actin WB)

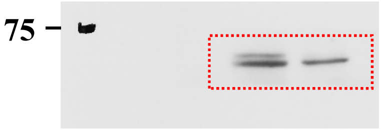

Appendix Fig S2D
(Roscovitine)
(p-Tfcp2l1 WB)

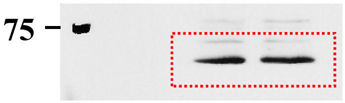

Appendix Fig S2D
(Roscovitine)
(t-Tfcp2l1 WB)

Appendix Fig S2

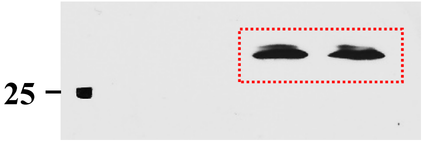

Appendix Fig S2D
(CDK1 WB)
(Roscovitine)

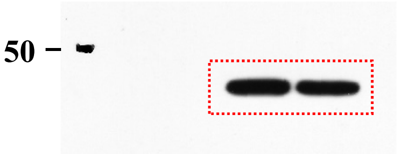

Appendix Fig S2D
(β-actin WB)
(Roscovitine)

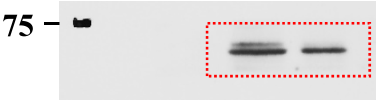

Appendix Fig S2D
(p-Tfcp2l1 WB)
(shCdk1)

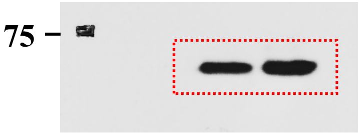

Appendix Fig S2D
(t-Tfcp2l1 WB)
(shCdk1)

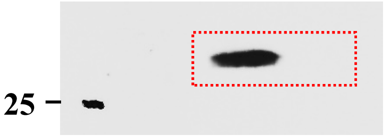

Appendix Fig S2D
(CDK1 WB)
(shCdk1)

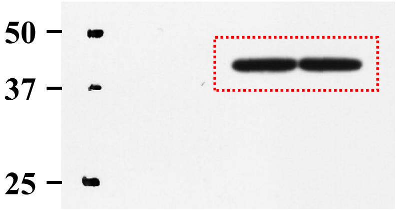

Appendix Fig S2D
(β-actin WB)
(shCdk1)

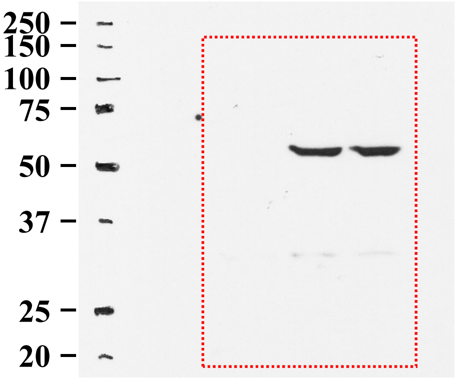

Appendix Fig S2E
(p-Tfcp2l1 WB)

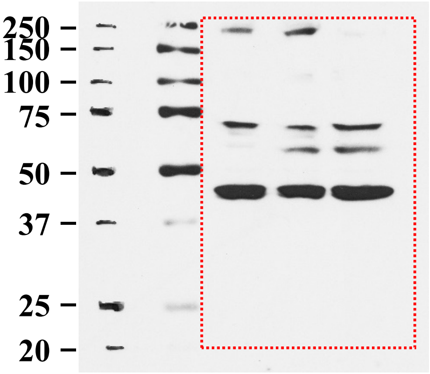

Appendix Fig S2E
(t-Tfcp2l1 WB)
