# Supplementary material for: Phosphorylation of TFCP2L1 by CDK1 is required for stem cell pluripotency and bladder carcinogenesis
Source: EMBO Mol Med. 2019 Nov 11;12(1):e10880. doi: 10.15252/emmm.201910880 (PMC6949511; doi:10.15252/emmm.201910880)
Supplement: Supplementary file 5 — Source Data for Expanded View and Appendix [file EMMM-12-e10880-s012.zip › Heoetal_Source_data_EV_Appendix/Heoetal_Source_data_uncropped_Fig_EV1.pdf]

Fig EV1

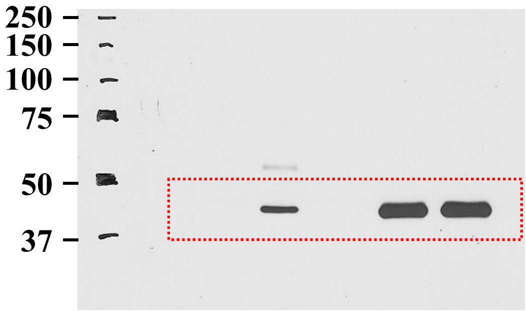

Fig EV1A
(Oct-4 WB)

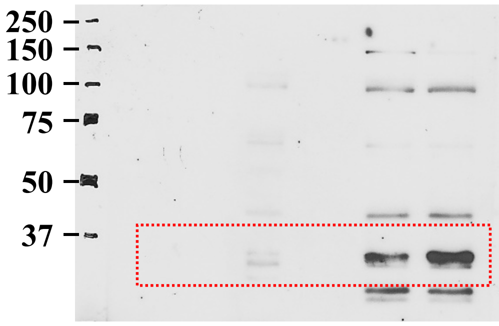

Fig EV1A
(SOX-2 WB)

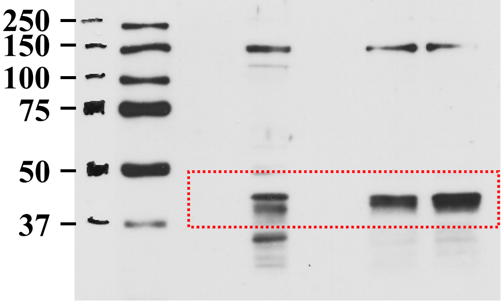

Fig EV1A
(Nanog WB)

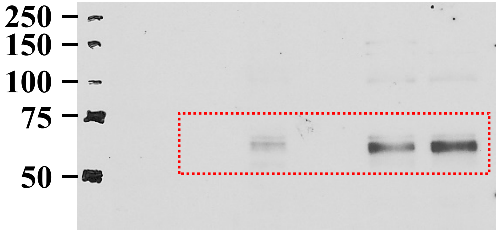

Fig EV1A
(Klf4 WB)

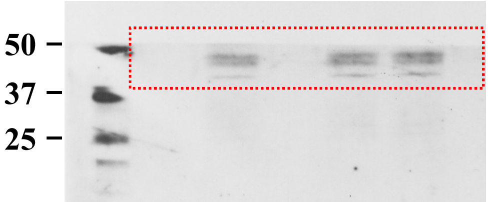

Fig EV1A
(Klf2 WB)

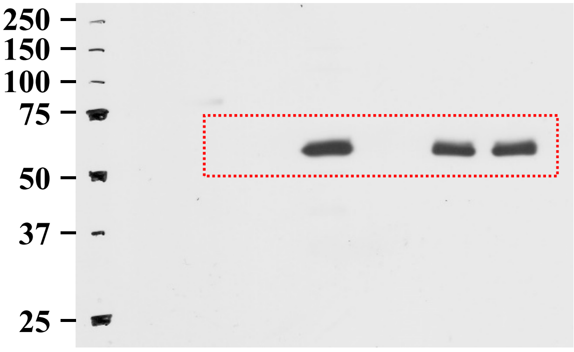

Fig EV1A
(Hdac1 WB)

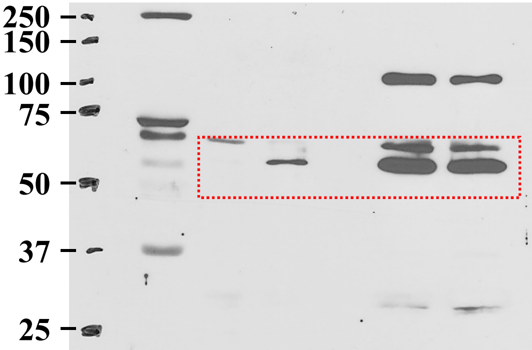

Fig EV1A
(Hdac2 WB)

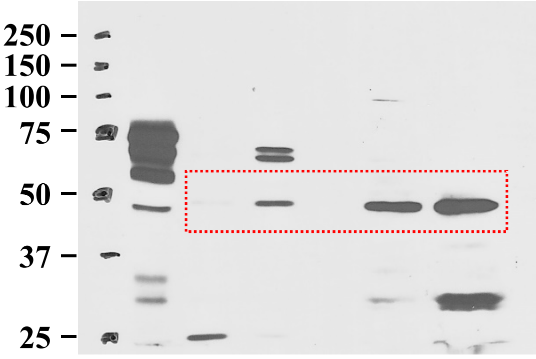

Fig EV1A
(Hdac3 WB)

Fig EV1

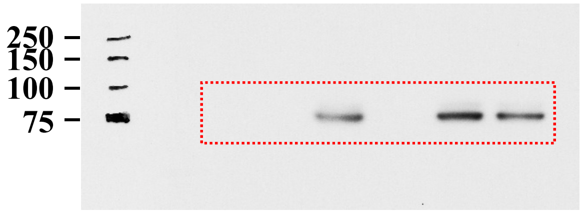

Fig EV1A
(Mta1 WB)

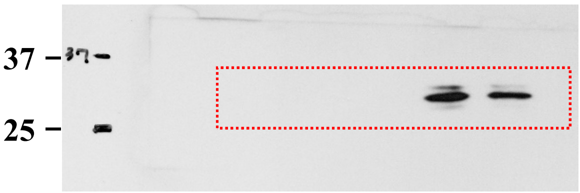

Fig EV1A
(Mbd3 WB)

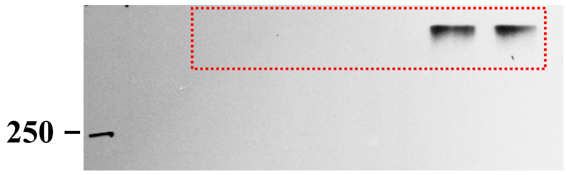

Fig EV1A
(Trrap WB)

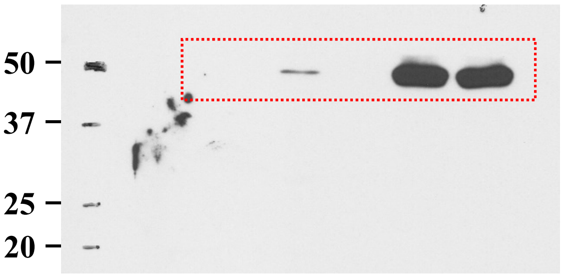

Fig EV1A
(Ruvbl2 WB)

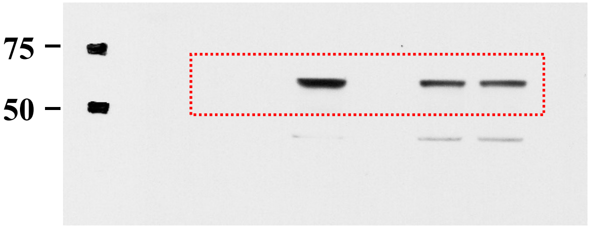

Fig EV1A
(Tip60 WB)

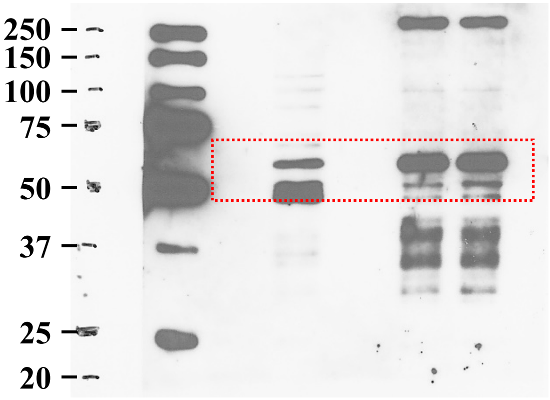

Fig EV1A
(DNMAP-1 WB)

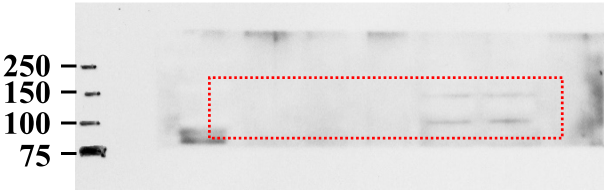

Fig EV1A
(pCAF WB)

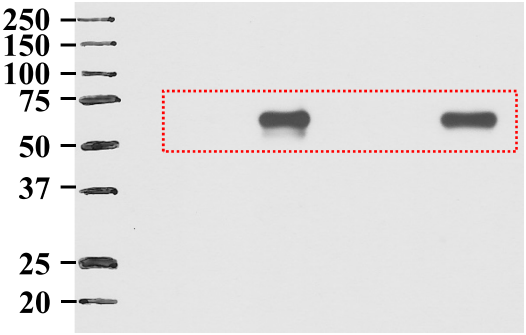

Fig EV1A
(Flag WB)
