# Supplementary material for: Phosphorylation of TFCP2L1 by CDK1 is required for stem cell pluripotency and bladder carcinogenesis
Source: EMBO Mol Med. 2019 Nov 11;12(1):e10880. doi: 10.15252/emmm.201910880 (PMC6949511; doi:10.15252/emmm.201910880)
Supplement: Supplementary file 5 — Source Data for Expanded View and Appendix [file EMMM-12-e10880-s012.zip › Heoetal_Source_data_EV_Appendix/Heoetal_Source_data_uncropped_Fig_EV2.pdf]

Fig EV2

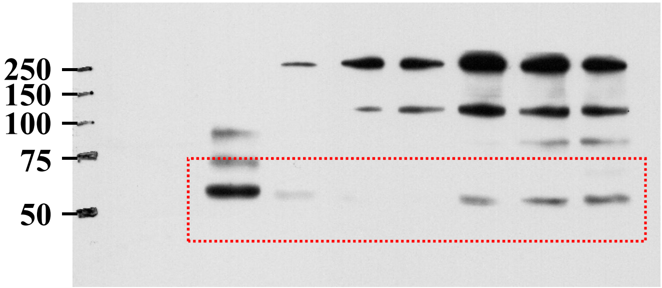

Fig EV2A
(p-Tfcp2l1 WB)

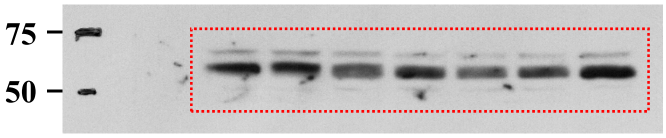

Fig EV2A
(t-Tfcp2l1 WB)

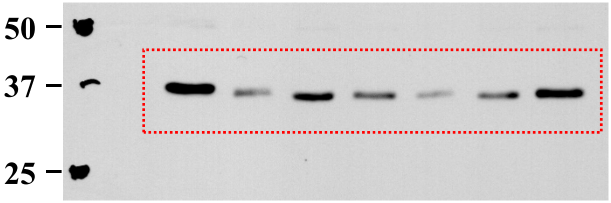

Fig EV2A
(SOX-2 WB)

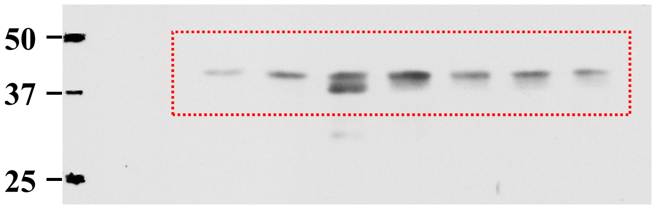

Fig EV2A
(Nanog WB)

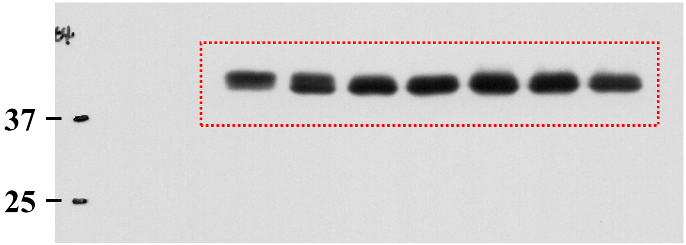

Fig EV2A
(Oct-4 WB)

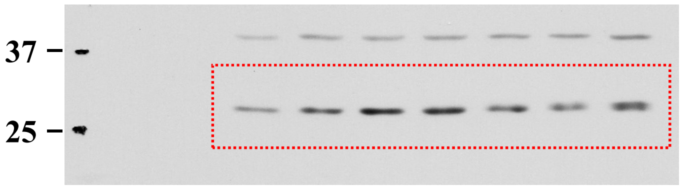

Fig EV2A
(CDK1 WB)

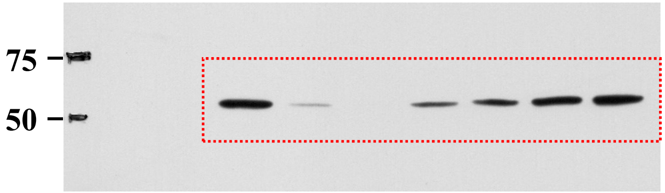

Fig EV2A
(Cyclin B WB)

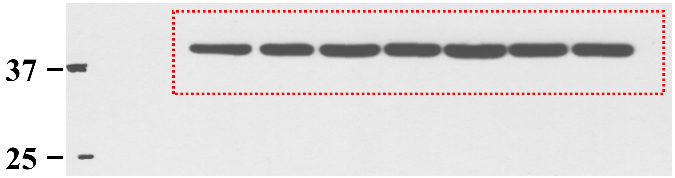

Fig EV2A
(β-actin WB)

Fig EV2

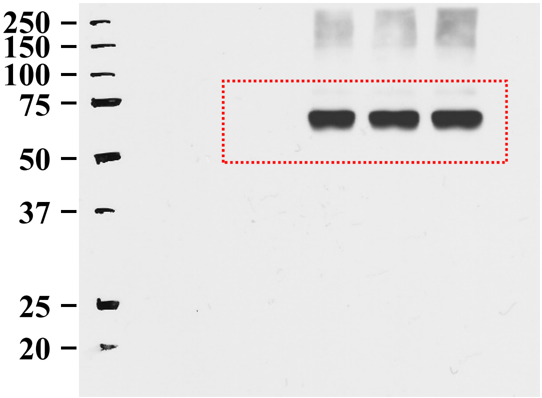

Fig EV2G
(Flag WB)

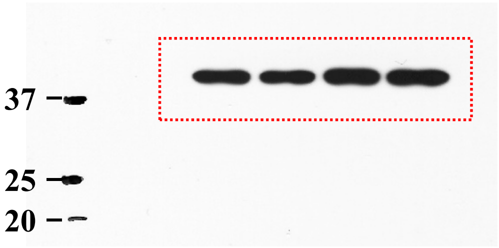

Fig EV2G
(β-actin WB)
