# Supplementary material for: Phosphorylation of TFCP2L1 by CDK1 is required for stem cell pluripotency and bladder carcinogenesis
Source: EMBO Mol Med. 2019 Nov 11;12(1):e10880. doi: 10.15252/emmm.201910880 (PMC6949511; doi:10.15252/emmm.201910880)
Supplement: Supplementary file 7 — Source Data for Figure 1 [file EMMM-12-e10880-s005.zip › Heoetal_Source_data_fig1/Heoetal_Source_data_uncropped_Fig1.pdf]

Fig 1

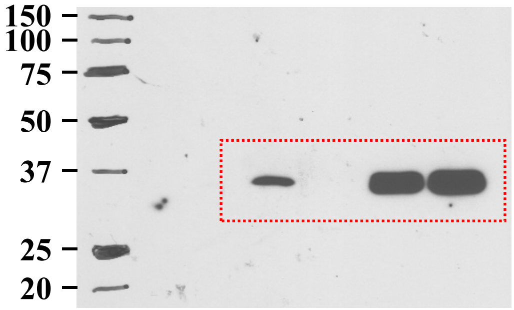

Fig 1C
(HA WB)

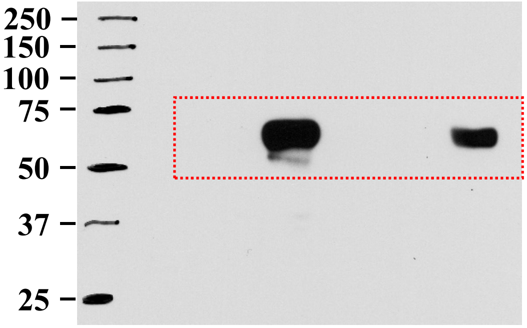

Fig 1C
(Flag WB)

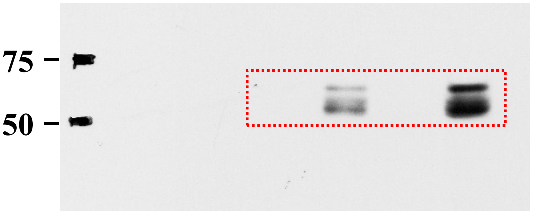

Fig 1C
(Tfcp2l1 WB)

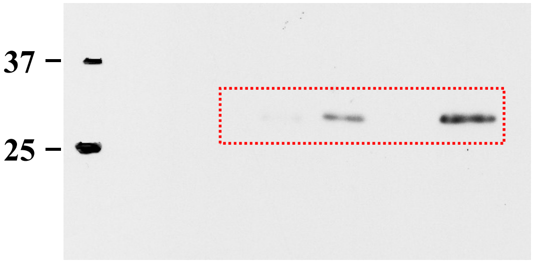

Fig 1C
(CDK1 WB)

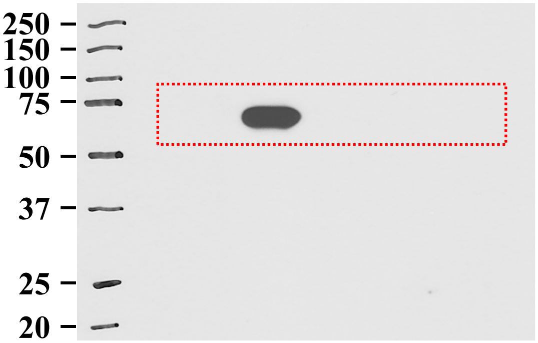

Fig 1D
(p-Thr WB)

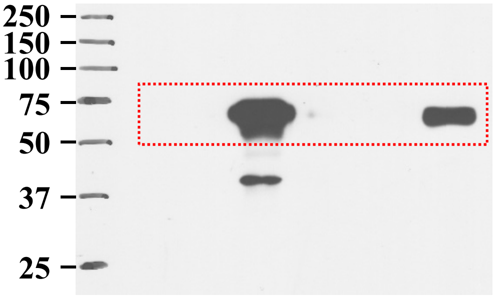

Fig 1D
(Flag WB)

Fig 1

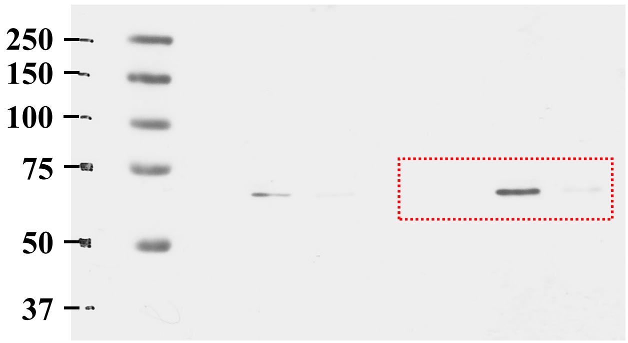

Fig 1F
(p-Thr WB)

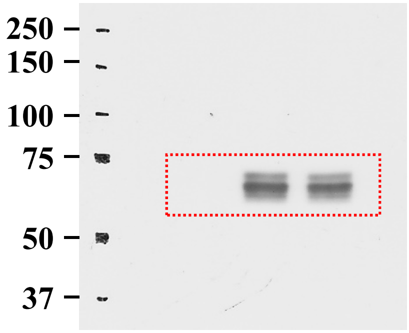

Fig 1F
(Flag WB)

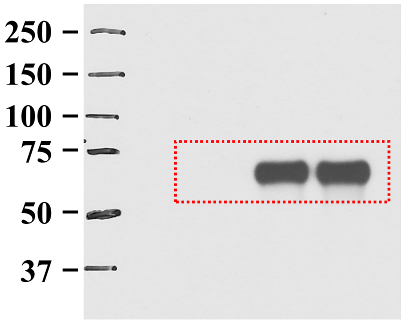

Fig 1F
(Flag WB)

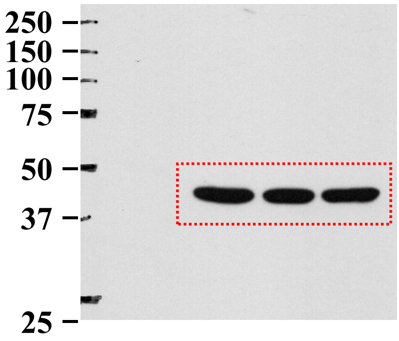

Fig 1F
(β-actin WB)

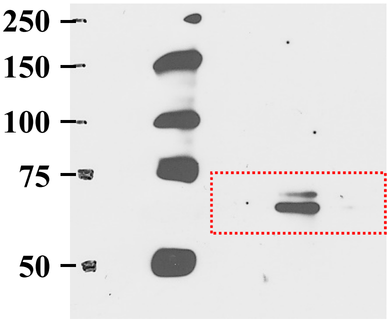

Fig 1G
(p-Thr WB)

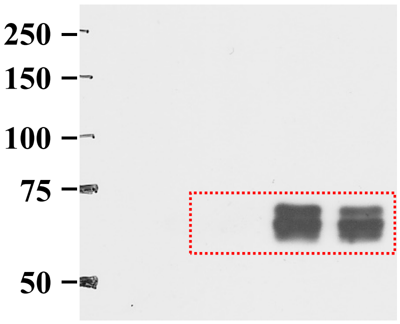

Fig 1G
(Flag WB)

Fig 1

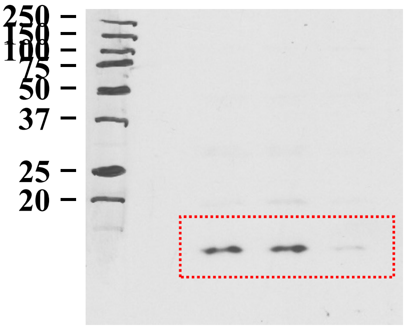

Fig 1G
(p-H3S10 WB)

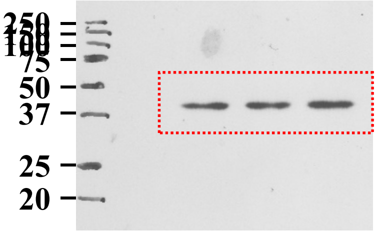

Fig 1G
(β-actin WB)

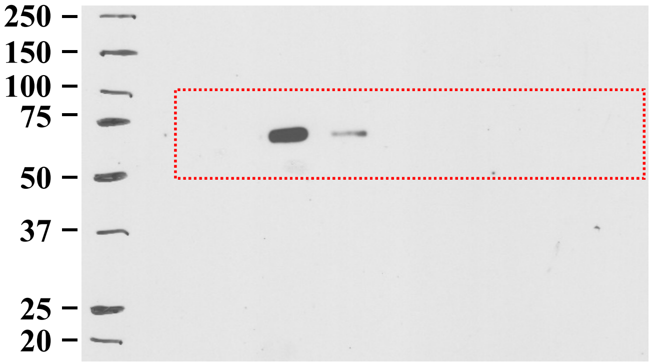

Fig 1H
(P-Thr WB)

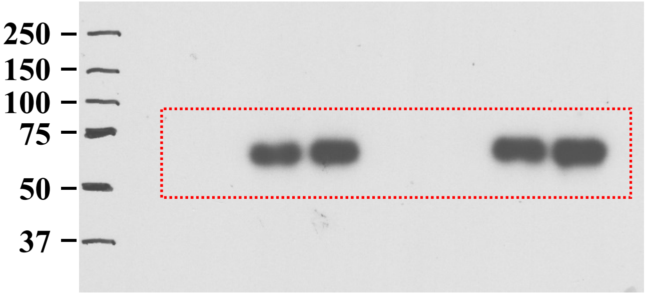

Fig 1H
(Flag WB)

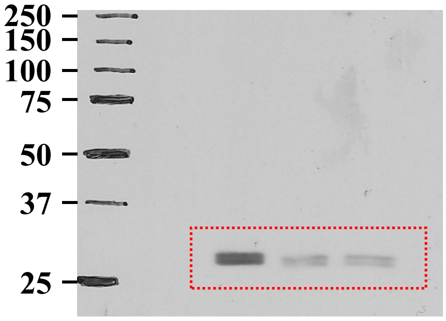

Fig 1H
(CDK1 WB)

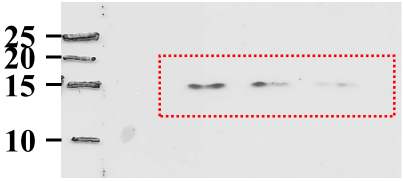

Fig 1H
(p-H3S10 WB)

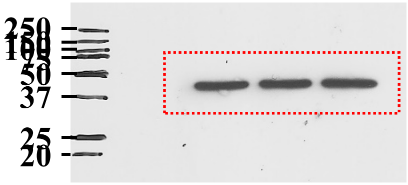

Fig 1H
(β-actin WB)
