# Supplementary material for: Phosphorylation of TFCP2L1 by CDK1 is required for stem cell pluripotency and bladder carcinogenesis
Source: EMBO Mol Med. 2019 Nov 11;12(1):e10880. doi: 10.15252/emmm.201910880 (PMC6949511; doi:10.15252/emmm.201910880)
Supplement: Supplementary file 11 — Source Data for Figure 5 [file EMMM-12-e10880-s009.zip › Heoetal_Source_data_fig5/Heoetal_Source_data_uncropped_Fig5.pdf]

Fig 5

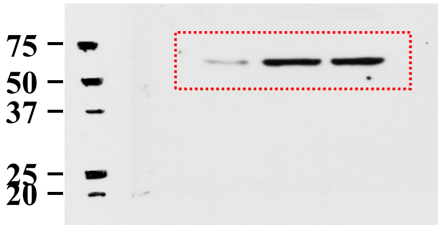

Fig 5A
(TFCP2L1 WB)

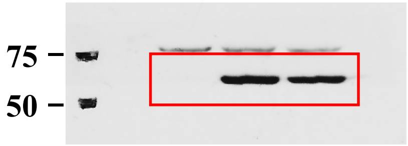

Fig 5A
(P-Tfcp2l1 WB)

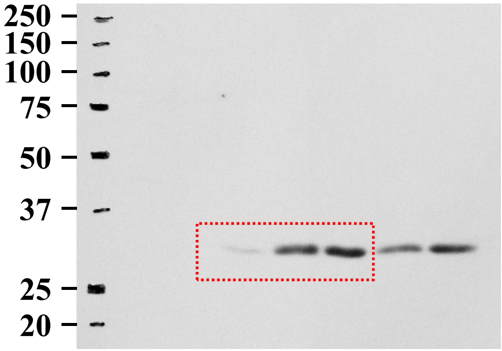

Fig 5A
(CDK1 WB)

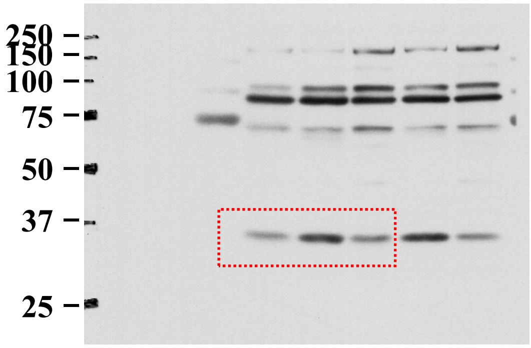

Fig 5A
(KLF2 WB)

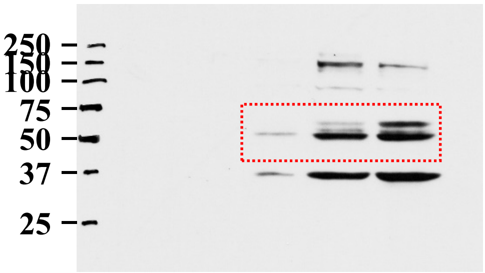

Fig 5A
(KLF4 WB)

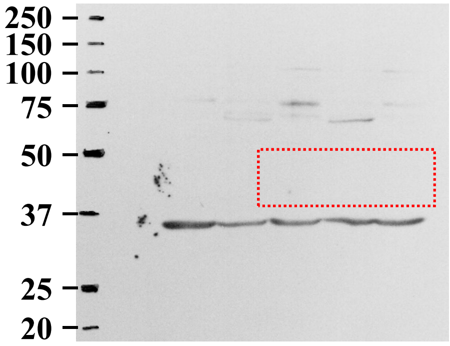

Fig 5A
(Oct-4 WB)

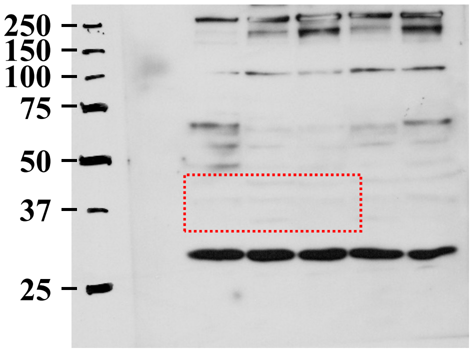

Fig 5A
(hNANOG WB)

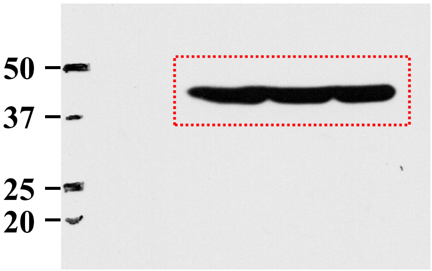

Fig 5A
(β-actin WB)

Fig 5

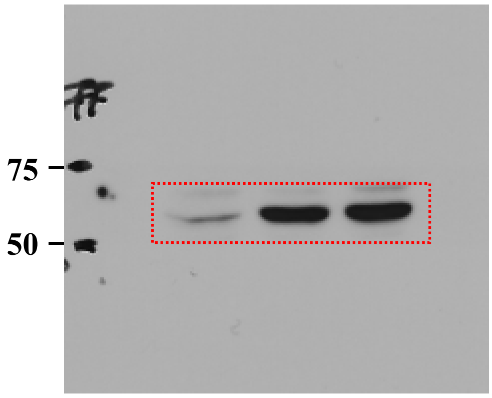

Fig 5D
(TFCP2L1 WB)

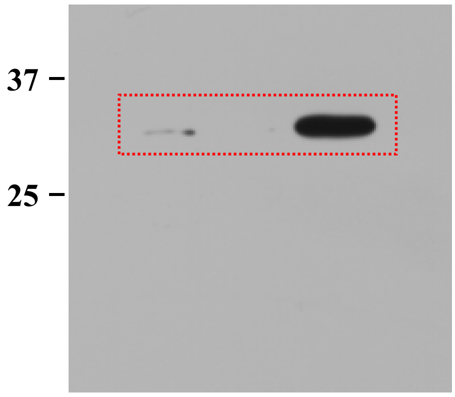

Fig 5D
(CDK1 WB)

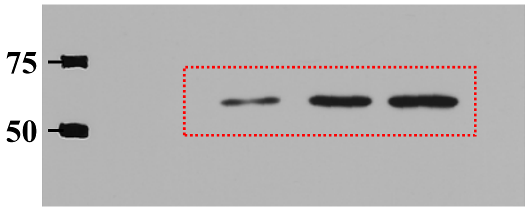

Fig 5D
(p-TFCP2L1 WB)

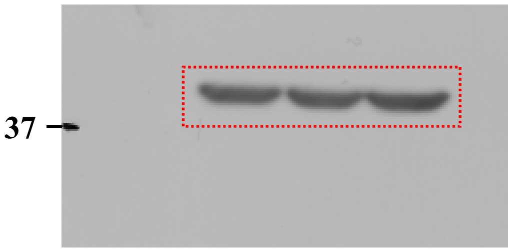

Fig 5D
(β-actin WB)

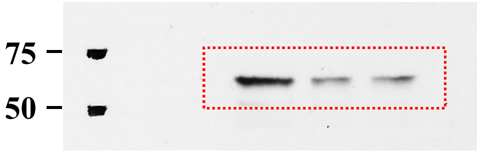

Fig 5D
(TFCP2L1 WB)

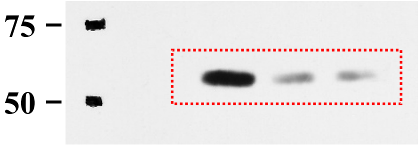

Fig 5D
(p-TFCP2L1 WB)

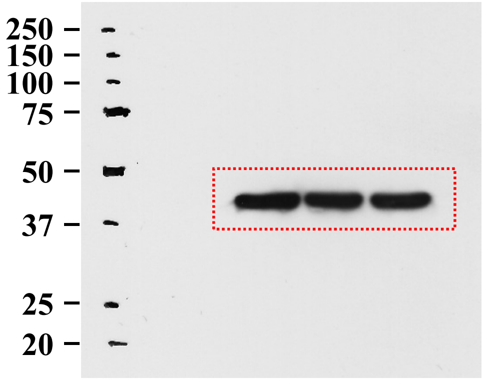

Fig 5D
(β-actin WB)

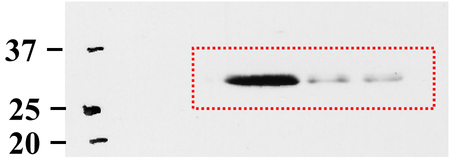

Fig 5D
(CDK1 WB)

Fig 5

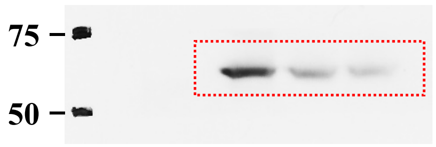

Fig 5D
(p-TFCP2L1 WB)

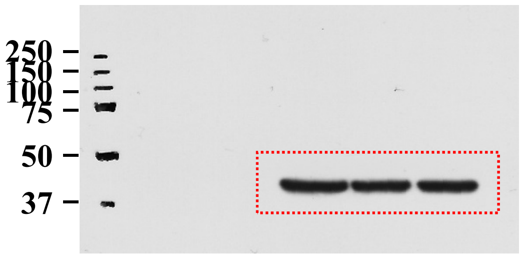

Fig 5D
(β-actin WB)
