# Supplementary material for: Phosphorylation of TFCP2L1 by CDK1 is required for stem cell pluripotency and bladder carcinogenesis
Source: EMBO Mol Med. 2019 Nov 11;12(1):e10880. doi: 10.15252/emmm.201910880 (PMC6949511; doi:10.15252/emmm.201910880)
Supplement: Supplementary file 12 — Source Data for Figure 6 [file EMMM-12-e10880-s010.zip › Heoetal_Source_data_fig6/Heoetal_Source_data_uncropped_Fig6.pdf]

Fig 6

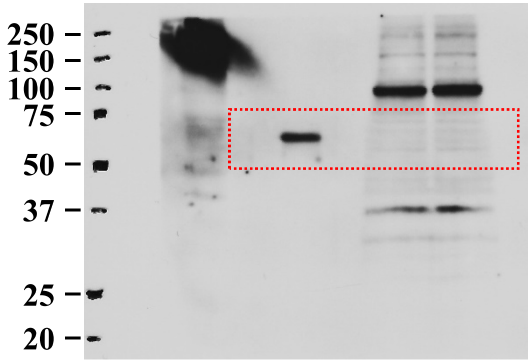

Fig 6D
(p-Thr WB)
(IP : Flag)

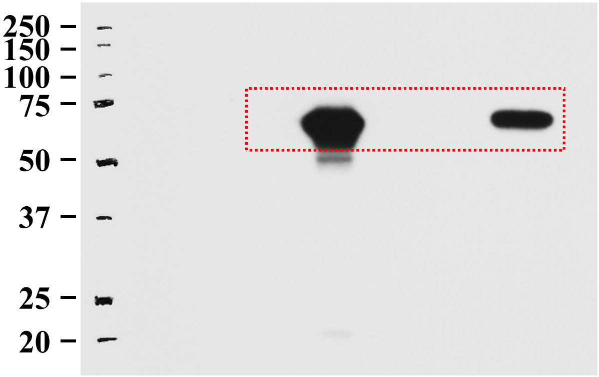

Fig 6D
(Flag WB)
(IP : Flag)

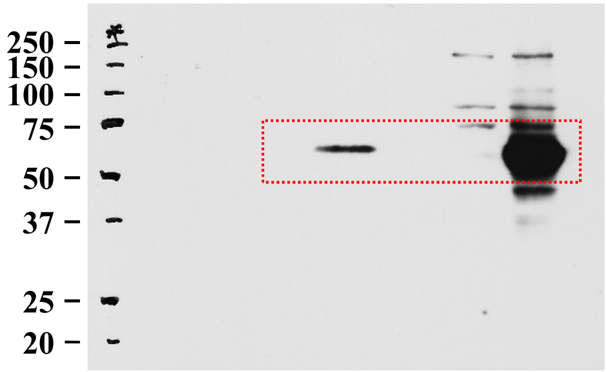

Fig 6D
(Flag WB)
(IP : p-Thr)

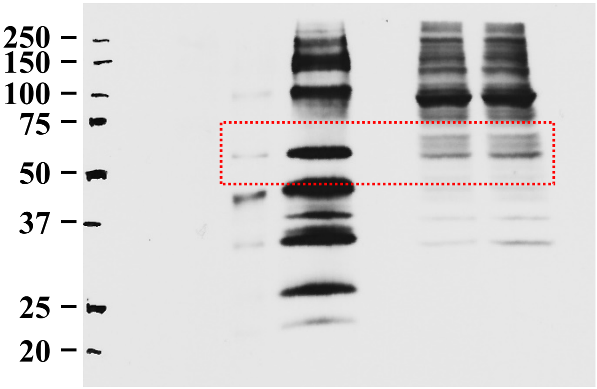

Fig 6D
(p-Thr WB)
(IP : p-Thr)

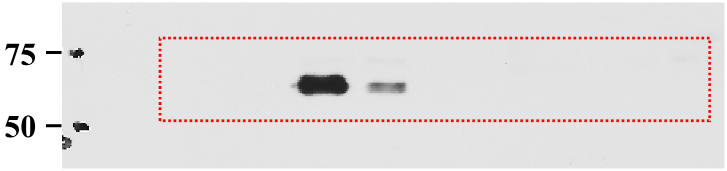

Fig 6E
(p-Thr WB)

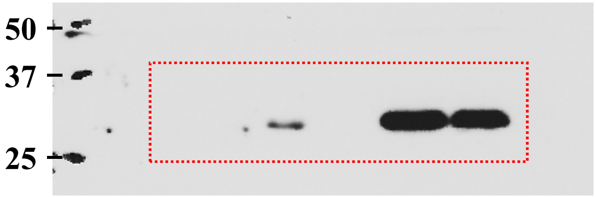

Fig 6F
(HA WB)

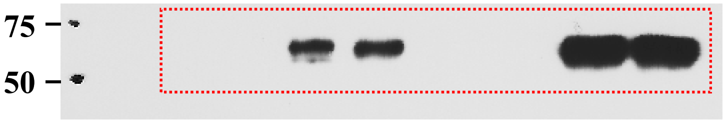

Fig 6E
(Flag WB)

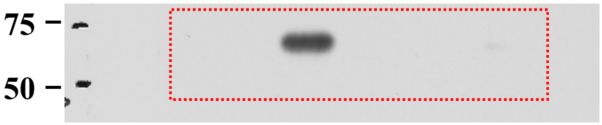

Fig 6F
(Flag WB)
