# Supplementary figures and images for: Phosphorylation of TFCP2L1 by CDK1 is required for stem cell pluripotency and bladder carcinogenesis
Source: EMBO Mol Med. 2019 Nov 11;12(1):e10880. doi: 10.15252/emmm.201910880 (PMC6949511; doi:10.15252/emmm.201910880)

**Fig 7**

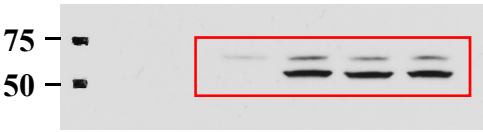

**Fig 7A**  
**(TFCP2L1 WB)**

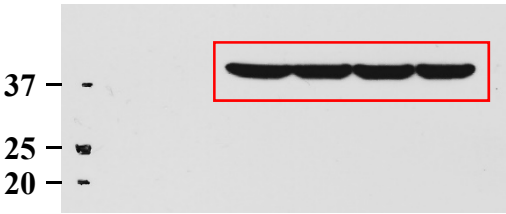

**Fig 7A**  
**( $\beta$ -actin WB)**

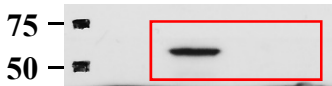

**Fig 7A**  
**(TFCP2L1 WB)**

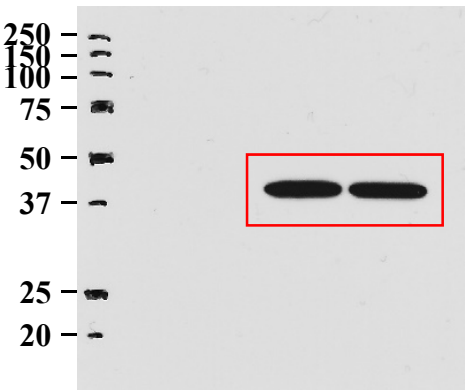

**Fig 7A**  
**( $\beta$ -actin WB)**

Supplement: Supplementary file 13 — Source Data for Figure 7 [file EMMM-12-e10880-s011.zip › Heoetal_Source_data_fig7/Heoetal_Source_data_uncropped_Fig7.pdf]
